# Microbiota of Cow’s Milk; Distinguishing Healthy, Sub-Clinically and Clinically Diseased Quarters

**DOI:** 10.1371/journal.pone.0085904

**Published:** 2014-01-20

**Authors:** Georgios Oikonomou, Marcela Lucas Bicalho, Enoch Meira, Rodolfo Elke Rossi, Carla Foditsch, Vinicius Silva Machado, Andre Gustavo Vieira Teixeira, Carlos Santisteban, Ynte Hein Schukken, Rodrigo Carvalho Bicalho

**Affiliations:** Department of Population Medicine and Diagnostic Sciences, College of Veterinary Medicine, Cornell University, Ithaca, New York, United States of America; University of Alberta, Canada

## Abstract

The objective of this study was to use pyrosequencing of the 16S rRNA genes to describe the microbial diversity of bovine milk samples derived from clinically unaffected quarters across a range of somatic cell counts (SCC) values or from clinical mastitis, culture negative quarters. The obtained microbiota profiles were used to distinguish healthy, subclinically and clinically affected quarters. Two dairy farms were used for the collection of milk samples. A total of 177 samples were used. Fifty samples derived from healthy, culture negative quarters with a SCC of less than 20,000 cells/ml (group 1); 34 samples derived from healthy, culture negative quarters, with a SCC ranging from 21,000 to 50,000 cells/ml (group 2); 26 samples derived from healthy, culture negative quarters with a SCC greater than 50,000 cells/ml (group 3); 34 samples derived from healthy, culture positive quarters, with a SCC greater than 400,000 (group 4, subclinical); and 33 samples derived from clinical mastitis, culture negative quarters (group 5, clinical). Bacterial DNA was isolated from these samples and the 16S rRNA genes were individually amplified and pyrosequenced. All samples analyzed revealed great microbial diversity. Four bacterial genera were present in every sample obtained from healthy quarters (*Faecalibacterium* spp., unclassified Lachnospiraceae, *Propionibacterium* spp. and *Aeribacillus* spp.). Discriminant analysis models showed that samples derived from healthy quarters were easily discriminated based on their microbiota profiles from samples derived from clinical mastitis, culture negative quarters; that was also the case for samples obtained from different farms. *Staphylococcus spp.* and *Streptococcus spp.* were among the most prevalent genera in all groups while a general multivariable linear model revealed that *Sphingobacterium* and *Streptococcus* prevalences were associated with increased 10 log SCC. Conversely, *Nocardiodes* and *Paenibacillus* were negatively correlated, and a higher percentage of the genera was associated with a lower 10 log SCC.

## Introduction

Mastitis is a disease in dairy cows with a high incidence and prevalence and arguably one of the most important for the dairy industry worldwide. Mastitis causes severe economic losses due to reduced milk production, discarded milk, lower conception rates, premature culling, and treatment costs [1]–[3]. Somatic cell counts (SCC) have been extensively used to distinguish healthy quarters from quarters with an inflammatory response most likely due to an intramammary infection [4], [5]. Bulk milk somatic cell counts are used as an overall indicator of milk quality [6].

Intramammary infections are currently defined as identified predominantly by aerobic culture, modified anaerobic and in some cases by molecular diagnostics [7], [8]. However, approximately 25% of clinical mastitis samples are culture negative or show no significant pathogens. Similarly, 30% of samples from cows or quarters with high SCC (subclinically affected quarters) were reported to be culture negative [9]. As expected, most quarters with a low SCC, usually defined as values below 200,000 cells/ml, are culture negative in aerobic and modified anaerobic culture. Although aerobic bacteria and *Mycoplasma* spp. that cause mastitis have been extensively studied, studies on the role of the normal microbiota of bovine milk are still scarce [10], [11]. Empirical evidence for the importance of native bacterial populations within the mammary gland, comes from the reports of clinical *Escherichia coli* mastitis outbreak following “blitz” therapy (simultaneous antibacterial treatment of all infected mammary glands within a herd to eliminate *Streptococcus agalactiae* infection from a herd) [12]. More detailed evidence of a native bacterial population in healthy quarters was previously reported by our research team [10].

In this previous report, we argued that sequencing and analysis of hyper variable regions within the 16S rRNA gene provided relatively rapid and cost-effective methods for assessing bacterial diversity in mammary secretion from healthy and affected cows [10]. Hunt et al. (2011) [13] used barcoded pyrosequencing to characterize the diversity of bacterial communities in milk samples taken from human mammary glands and showed that this technique identified a much greater diversity of bacteria in milk than what had previously been reported in culture-independent studies. Cabrera-Rubio et al. (2012) [14] characterized bacterial communities in human milk at 3 different time points in healthy mothers who varied in body mass index, weight gain and mode of delivery, and reported a diverse array of microorganisms. Their data also indicated significant changes in milk microbiome composition and diversity through lactation and suggested that the milk microbiome is compositionally distinct from any other ecological niche in the human body and that it is not a simple reflection of the microbiome of the skin.

As indicated, this very same 16S based barcoded pyrosequencing was recently used by our group [10] to investigate bacterial DNA diversity in milk samples of a large population of mastitic and a small number of healthy bovine mammary glands. The obtained sequences were compared to results obtained by classical bacterial culture. Generally, the dominant species identified in the obtained sequences was also the species identified in bacterial culture in clinically affected glands. We also demonstrated that the microbiota of samples derived from low SCC and aerobic culture negative glands was clearly different from the microbiota of the mastitic samples, but the number of samples from low SCC glands was relatively small and did not provide a full picture of the normal microbiota of the bovine mammary gland across a range of SCC values.

The objective of this study was to use pyrosequencing of the 16s rRNA gene to describe the microbial diversity of bovine milk samples derived from clinically unaffected quarters across a range of SCC values and to use the obtained microbiota profiles to distinguish healthy, subclinically and clinically affected glands.

## Materials and Methods

### Sample collection

Two large dairy farms located near Ithaca NY, were used for the collection of milk samples after the farms’ owners gave their permission. Milk samples were collected during the morning milking by members of the research team. Sampling methods followed standard recommendations by the National Mastitis Council and were carried out with great diligence and emphasis on pre-sampling disinfection of teat-ends and hygiene during sampling. The research protocol was reviewed and approved by the Institutional Animal Care and Use Committee of Cornell University (Protocol number: 2009-0070). Cows with no previous history of clinical mastitis in the same lactation, and with two consecutive monthly SCC values lower than 100,000 cells/ml immediately prior to sample collection, were eligible to be included in the study. Cows were selected across a range of SCC values. As the mammary gland of a cow has four compartments, the unit of sampling in bovine studies is the so-called mammary gland quarter. In total, 560 quarter milk samples were obtained from 150 cows. Samples were kept on ice and transferred to our laboratory at the College of Veterinary Medicine, Cornell University. Samples were thoroughly mixed before aliquotting. One aliquot from each sample was used for SCC analysis, performed at the Dairy One Cooperative Inc. milk laboratory (Ithaca, NY). A second aliquot was used for aerobic bacterial culture. Using sterile swabs a small quantity of each milk sample was applied on SPC agar plates and aerobically incubated for 24 hours at 37°C.

After this initial screening, a third aliquot of 300 milk samples (292 SPC culture negative samples and 8 SPC culture positive with a SCC greater than 400,000) were used for full aerobic bacteriological culture, performed at the ISO 17025 accredited Quality Milk Production Services (QMPS) laboratory at Cornell University. Approximately 0.01 ml of each milk sample was inoculated using cotton swabs on trypticase soy agar plates containing 5% sheep blood and 0.1% esculin (bioMerieux, INC. Durham, NC 27704-0969 USA) and incubated aerobically at 37°C. Bacterial growth was identified after 24 and 48 h of incubation according to National Mastitis Council standards. Briefly, *Staphylococcus aureus* and *Staphylococcus* spp. were identified by haemolytic pattern and tube coagulate test. *Streptococcus dysgalactiae, Streptococcus uberis* and *Streptococcus* spp. were differentiated by presence or absence of esculin hydrolysis, Lancefield group C typing (PathoDx strep grouping latex agglutination test, Remel), and growth or growth inhibition on Bile Esculin Azide Agar (Enterococcosel™, Becton, Dickinson). *Escherichia coli* and *Klebsiella* spp. were identified using morphologic characteristics of colonies on MacConkey agar, production of indole, motility, and utilization of citrate. *Trueperella pyogenes* was identified by colonial characteristic, presence of complete hemolysis and Gram stain. No *Mycoplasma* culture or anaerobic culture was performed on the samples as both farms had no recent history of *Mycoplasma* mastitis.

Based on SCC and aerobic culturing results 144 milk samples (culture negative samples with a SCC ranging from 5,000 to 2,754,000 cells/ml and culture positive samples with a SCC greater than 400,000 cells/ml) were selected for further analysis of their microbial diversity. For comparison purposes, microbial diversity in 33 milk samples obtained from quarters that showed signs of clinical mastitis and that were aerobic culture negative and were not subjected to cell counting were included in the study [10]. Therefore, a total of 177 milk samples were eventually used for this study. In detail: 50 samples derived from clinically healthy, culture negative quarters with a SCC of less than 20,000 cells/ml (group 1, healthy low SCC); 34 samples derived from clinically healthy, culture negative quarters, with a SCC ranging from 21,000 to 50,000 cells/ml (group 2, healthy medium SCC); 26 samples derived from healthy, culture negative quarters with a SCC greater than 50,000 cells/ml (group 3, healthy higher SCC); 34 samples derived from clinically healthy, culture positive quarters, with a SCC greater than 400,000 cells/ml (group 4, subclinical); and 33 samples derived from clinical mastitis, culture negative quarters (group 5, clinical). The SCC cut-offs were established based on previous studies on SCC values in healthy quarters [4], [15].

### DNA extraction

From a fourth aliquot of the selected samples, one ml of milk was centrifuged for 10 min at room temperature at 13,200 rpm (16,100 rcf) in an Eppendorf 5415R centrifuge. The supernatant was discarded and the remaining pellet was resuspended in 400 µl of nuclease-free water. Isolation of genomic DNA was then performed by using a QIAamp DNA minikit (Qiagen) according to the manufacturer’s instructions, except that 400 µg of lysozyme was added to the bacterial suspension and incubated for 12 h at 56°C to maximize bacterial DNA extraction. DNA concentration and purity were evaluated by optical density using a NanoDrop ND-1000 spectrophotometer (NanoDrop Technologies, Rockland, DE, USA) at wavelengths of 230, 260 and 280 nm.

### PCR amplification of the V1-2 region of bacterial 16S rRNA genes

The 16S rRNA genes were individually amplified from each sample using a composite pair of primers containing a unique 10-base barcode, which was used to tag the PCR products from the respective samples. The forward primer was 5′-**CGTATCGCCTCCCTCGCGCCATCAG**NNNNNNNNNNTC *AGAGTTTGATCCTGGCTCAG*-3′: the bold sequence is the GS FLX Titanium Primer A, and the italicized sequence is the universal broadly conserved bacterial primer 27F. The reverse primer was 5′-**CTATGCGCCTTGCCAGCCCGCTCAG**NNNNNNNNNN CA *TGCTGCCTCCCGTAGGAGT*-3′: the bold sequence is the GS FLX Titanium Primer B, and the italicized sequence is the broad-range bacterial primer 338R. The chosen primers are widely used in microbial diversity studies and the amplified region (V1–V2) is considered optimal for phylogenetic analysis from pyrosequencing reads [16], [17]. The expected amplicon size according to primers used was approximately 300 bp. The sequence NNNNNNNNNN, which is identical in the forward and reverse primer of each pair, designates the unique 10-base barcode used to tag each PCR product. A two-base linker sequence (underlined) was inserted between the barcode and the template-specific sequence to help diminish any effect the composite primer might have on the efficiency of the amplifications. The specific pair of primers used was checked against the bovine genome with NCBI primer-BLAST [18] and was not found to anneal with bovine DNA. The PCRs were carried out in triplicate 20-µl reactions containing 0.3 µM forward and reverse primers, using approximately 50 ng of template DNA and 10 µl HotStar Taq Plus Mix kit (Qiagen). A modified touchdown thermal cycling was used for amplification and consisted of initial denaturation at 95°C for 2 min, followed by 30 cycles of denaturation at 95°C for 30 sec, annealing (starting at 68oC and subsequently decreased by 2oC/2 cycles until it reached 58°C at which temperature the 20 remaining cycles were performed) for 30 sec, extension at 72°C for 60 sec, and a final extension at 72°C for 7 min. Replicate amplicons were pooled, purified with a QIAquick PCR Purification Kit (Qiagen), and visualized by electrophoresis using 1.2% (wt/vol) agarose gels stained with 0.5 µg/ml ethidium bromide before sequencing. Blank controls, in which no DNA was added to the reaction, were performed. In all cases these blank controls failed to produce visible PCR products; these samples were not analyzed further.

### Barcoded pyrosequencing of bacterial 16S rRNA genes

Amplicons were quantified using the Quant-iT PicoGreen dsDNA Assay Kit (Invitrogen) and combined in equimolar ratios into a single tube. Pyrosequencing of the samples was carried at the Cornell University Life Sciences Core Laboratories Center using Roche 454 GS-FLX System Titanium Chemistry.

### Sequences library analysis and statistical analysis

In the analysis of the obtained results, grouping into the five previously defined groups was frequently used.

The obtained FASTA sequences file was uploaded in the Ribosomal Database Project (RDP) pipeline initial processor that trimmed the 16S primers, tag sorted the sequences, and filtered out additional sequences of low-quality. Primers were removed from the sequences, zero N’s were allowed while sequences shorter than 150 bp were also removed. DECIPHER was used for chimera sequences identification [19]. RDP Classifier at the RDP’s Pyrosequencing Pipeline was used to assign 16S rRNA gene sequences of each sample to the new phylogenetically consistent higher-order bacterial taxonomy using an 80% confidence threshold, providing information regarding different genera prevalence in each sample [20].

Different genera prevalence in each sample derived from the above described analysis were used as covariates in stepwise discriminant analysis models that were built in JMP Pro (SAS Institute Inc. North Carolina). Variables were removed in a stepwise manner until only variables with a P value < 0.05 were retained in the final model. The five groups of samples were used as the categorical variable in these analyses. Discriminant analysis was performed using all 5 groups of milk samples. The same discriminant analysis model was also performed after excluding samples from clinical quarters (group 5) or using only groups 1–3, excluding samples from subclinical and clinical quarters. Discriminant analysis was also performed using only results of groups 1–3 and source farm as the categorical variable.

To identify genera that are associated with an increased or decreased immune response as measured by SCC in healthy quarters (groups 1–3), a generalized multivariable linear model was used to predict the 10 Log of quarter SCC. Percentages of the sequences for each of the predominant genera were used as predictor variables while at the same time correcting for herd effects.

The results produced from the initial processing FASTA files were also uploaded in the RDP’s aligner, which aligns the sequences using the INFERNAL aligner, a Stochastic Context Free Grammar (SCFG)-based, secondary-structure aware aligner [21], and then processed by the complete linkage clustering tool (that clustered the aligned sequences into OTUs). The cluster file that was obtained from the above described process was subsequently used for the evaluation of the samples richness and diversity through the estimation of Shannon and Chao1 indexes, again using the RDP pyrosequencing pipeline. The Shannon index is a nonparametric diversity index that combines estimates of richness (the total number of OTUs) and evenness (the relative abundance of OTUs). For example, communities with one dominant species have a low index, whereas communities with a more even distribution of species have a higher index. Chao1 is a nonparametric estimator of the minimum richness (number of OTUs) and is based on the number of rare OTUs (singletons and doublets) within a sample. The above described process was also followed using all sequences from each group of samples until a cluster file was obtained for each group of samples. This file was subsequently used to obtain rarefaction curves for each group of samples, again using the RDP pyrosequencing pipeline. A linear regression model was used to evaluate differences between diversity indexes (Chao1 and Shannon) for the five different groups of samples. The number of sequences analyzed per sample is known to affect the diversity indexes estimates and therefore was fitted in the model.

To facilitate a detailed species level analysis of the obtained sequences, the following steps were followed: 200 sequences from each sample of each group were randomly selected, using the random number function of Excel, and used to create a new FASTA sequence file. This file was then processed through the RDP pyrosequencing pipeline. The produced file was uploaded in the RDP’s aligner, which aligns the sequences using the INFERNAL aligner, a Stochastic Context Free Grammar (SCFG)-based, secondary-structure aware aligner, and then processed by the complete linkage clustering tool (that clustered the aligned sequences in OTU). Finally, the representative sequence function was used to create one representative sequence for each OTU. Eventually, a new file of representative sequences was created. The Basic Local Alignment Search Tool (BLASTn algorithm) from the National Center for Biotechnology Information (NCBI) web pages (http://www. ncbi.nlm.nih.gov/BLAST/) was then used to examine the nucleotide collection (EMBL/GenBank/DDBJ/PDB) databases for sequences with high similarity to these representative sequences [22]. As previously described, in more detail, the very same methodology was used for the analysis of the 33 clinical mastitis samples [10].

## Results

### Sequencing results, genera prevalences

Pyrosequencing of the 177 milk samples produced 795,511 sequences; the sequence size ranged from 48 to 1201 bp. Sequences obtained from this project were submitted to NCBI’s Sequence Read Archive (SRA accession number: SRP030032). A total of 248,162 sequences were eventually used for analyses by the RDP classifier after exclusion due to trimming and quality control, the size of the selected sequences ranged from 162 to 433 bp.

The thirty four samples from group 4 were found to be aerobic culture positive for *Staphylococcus* spp. (19 samples) or for *Streptococcus* spp. (15 samples).

**Figure 1:**
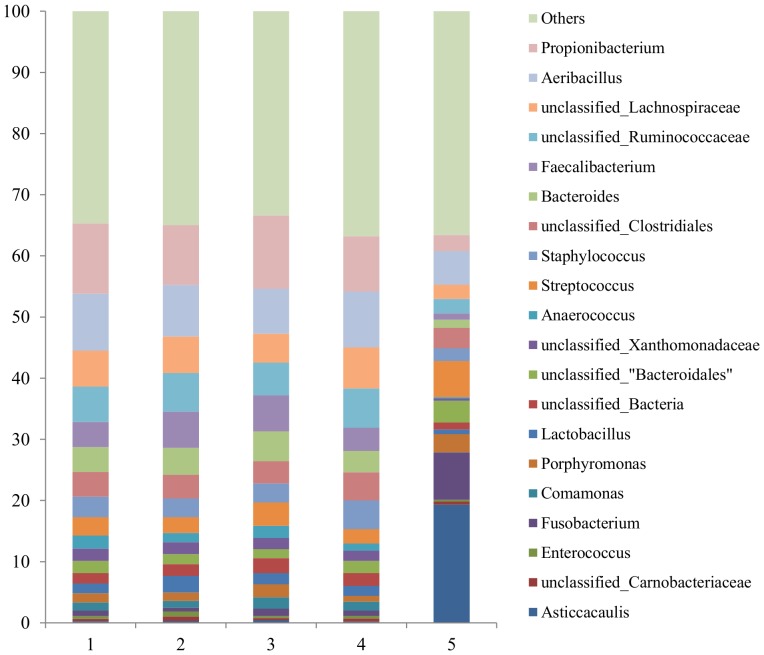
Average prevalence of the twenty most prevalent bacterial genera for each different group of samples (1 = healthy quarter, somatic cell count < 20.000; 2 = healthy quarter, somatic cell count ranged from 21,000 to 50,000; 3 = healthy quarter, somatic cell count >50,000; 4 = subclinical culture positive quarters, somatic cell count >400,000; 5 = mastitis quarter culture negative quarters).

In Figure 1 we present the average prevalence of the twenty most prevalent bacterial genera for each of the five groups of samples. Four of these bacterial genera (*Faecalibacterium*, unclassified *Lachnospiraceae*, *Propionibacterium* and *Aeribacillus*) were found to be present in all the studied samples derived from healthy quarters, regardless of their SCC or culture status.

Prevalences of known bacterial species in each of the five groups of milk samples are presented in Table 1. In this Table results of uncultured bacteria were combined into one category. These results are also presented in detail in Tables S1, S2, S3, S4, S5 in the supplemental material. The tables list the species-level information (with GenBank accession numbers and percentages of identity match) for each of the five groups of samples. Prevalence in these tables is defined as the number of sequences that were found to belong to each specific Operational Taxonomic Unit (OTU) out of the total number of sequences analysed for each group of samples.

**Table 1:** Prevalences of known bacterial species in each group of milk samples (1 = healthy quarter, somatic cell count < 20,000; 2 = culture negative quarter, somatic cell count ranged from 21,000 to 50,000; 3 = culture negative quarter, somatic cell count >50,000; 4 = non-clinical culture positive quarters, somatic cell count>400,000; 5 = clinical mastitis culture negative quarters).

Species	1	2	3	4	5
*Propionibacterium acnes*	11.37	9.05	13.26	8.35	2.76
*Geobacillus pallidus*	8.98	8.93	6.97	10.08	5.09
*Caulobacter leidyia*	0.00	0.36	0.35	0.00	20.55
*Streptococcus uberis*	2.03	1.42	1.88	1.22	4.82
Uncultured *Bacteroides*	0.63	2.03	2.71	1.26	0.00
*Staphylococcus epidermidis*	1.82	1.57	1.51	1.13	0.00
Uncultured *Bacteroides*	0.63	2.03	2.71	0.54	0.00
Uncultured *Fusobacteria*	0.00	0.00	0.00	0.00	5.49
Uncultured *Clostridiales*	0.65	0.00	2.29	1.99	0.00
Uncultured *Porphyromonas*	0.58	0.48	0.60	0.32	2.46
*Porphyromonas levii*	0.46	0.51	0.46	0.41	2.09
*Staphylococcus equorum*	0.58	0.52	0.52	0.46	1.40
*Lactobacillus johnsonii*	0.71	0.95	0.79	0.68	0.00
*Bacteroides heparinolyticus*	0.81	0.43	0.44	0.00	1.30
Uncultured *Firmicutes*	1.50	0.95	0.00	0.00	0.00
*Staphylococcus chromogenes*	0.00	0.00	0.00	2.27	0.00
*Clostridiales bacterium*	0.00	2.10	0.00	0.00	0.00
*Fusobacterium necrophorum*	0.47	0.39	0.71	0.43	0.00
*Bacteroides fragilis*	0.53	0.54	0.52	0.38	0.00
*Bacteroides vulgatus*	0.94	1.00	0.00	0.00	0.00
*Prevotella*	0.00	0.00	0.00	1.14	0.70
Uncultured *Proteobacterium*	0.00	0.00	1.58	0.00	0.00
*Lactobacillus acidophilus*	0.00	0.51	0.54	0.30	0.00
*Comamonas kerstersii*	1.32	0.00	0.00	0.00	0.00
*Lactobacillus reuteri*	0.00	0.75	0.00	0.48	0.00
*Ureaplasma diversum*	0.00	0.00	0.00	0.00	1.23
*Bacteroides coprophilus*	1.13	0.00	0.00	0.00	0.00
*Acidovorax*	0.00	1.12	0.00	0.00	0.00
*Kocuria*	0.43	0.00	0.66	0.00	0.00
*Paenibacillus borealis*	0.00	0.00	0.00	0.00	1.03
Uncultured *Prevotella*	0.00	0.00	0.00	0.00	0.96
Uncultured *Helcococcus*	0.00	0.39	0.00	0.54	0.00
Uncultured *Lachnospiraceae*	0.00	0.88	0.00	0.00	0.00
*Corynebacterium falsenii*	0.00	0.34	0.00	0.00	0.53
*Enterococcus faecalis*	0.33	0.49	0.00	0.00	0.00
*Faecalibacterium prausnitzii*	0.82	0.00	0.00	0.00	0.00
*Rhodanobacter*	0.35	0.00	0.41	0.00	0.00
*Staphylococcus aureus*	0.32	0.39	0.00	0.00	0.00
Uncultured *Staphylococcus*	0.00	0.00	0.38	0.00	0.33
Uncultured *Halomonas*	0.35	0.33	0.00	0.00	0.00
*Histophilus somni*	0.00	0.00	0.00	0.00	0.66
*Bacteroides denticanum*	0.00	0.31	0.35	0.00	0.00
*Bacillus*	0.00	0.00	0.00	0.00	0.63
*Ochrobactrum pseudogrignonense*	0.00	0.00	0.00	0.00	0.57
*Staphylococcus*	0.00	0.00	0.00	0.54	0.00
*Helcococcus ovis*	0.00	0.00	0.00	0.00	0.53
*Clostridium*	0.00	0.52	0.00	0.00	0.00
*Piscibacillus*	0.00	0.00	0.00	0.49	0.00
*Trueperella pyogenes*	0.00	0.00	0.00	0.00	0.43
*Delftia spp.*	0.00	0.00	0.00	0.00	0.40
Uncultured *Clostridia*	0.00	0.00	0.00	0.40	0.00
*Escherichia coli*	0.00	0.00	0.00	0.00	0.37
*Mycoplasma bovigenitalium*	0.00	0.00	0.00	0.00	0.37
Uncultured *Ruminococcaceae*	0.00	0.00	0.00	0.00	0.37
*Rhodanobacter terrae*	0.00	0.36	0.00	0.00	0.00
*Brevibacillus parabrevis*	0.00	0.00	0.00	0.00	0.33
*Uncultured Bacteroidetes*	0.00	0.00	0.00	0.00	0.33
*Xanthomonas campestris*	0.00	0.00	0.00	0.00	0.33
*Geobacillus*	0.00	0.00	0.00	0.32	0.00
Uncultured *bacteria*	14.19	16.97	17.21	12.32	11.86

Prevalence of all bacteria that were not identified at species level (uncultured bacteria) were summed and presented in the last line of the table.

### Discriminant analysis results

**Figure 2:**
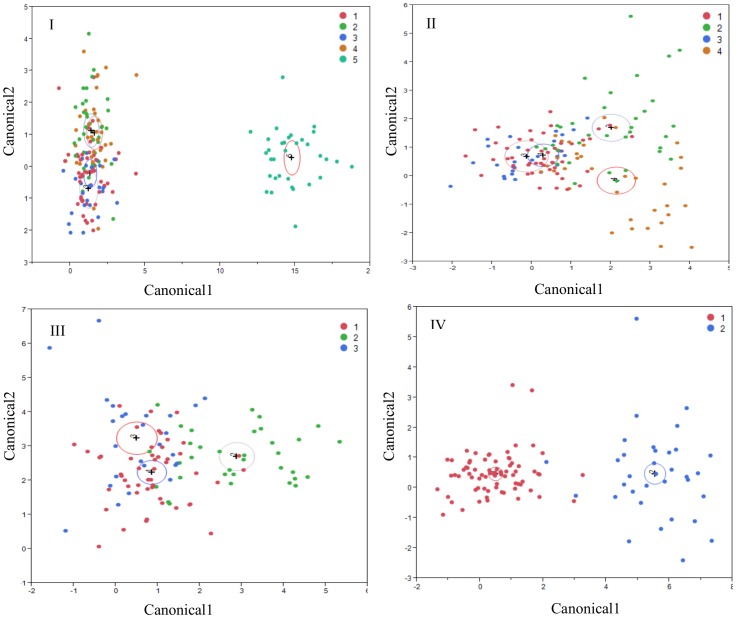
Discriminant analyses of milk samples microbiome. I. Discriminant analysis that was performed using all 5 groups of milk samples (1 = healthy quarter, somatic cell count < 20,000; 2 = healthy quarter, somatic cell count ranged from 21,000 to 50,000; 3 = healthy quarter, somatic cell count >50,000; 4 = subclinical culture positive quarters, somatic cell count>400,000; 5 = clinical mastitis culture negative quarters). II. Discriminant analysis that was performed after excluding samples derived from clinical mastitis, culture negative quarters (group 1–4 included). III. Discriminant analysis performed using groups 1–3. IV. Discriminant analysis of milk microbiome from healthy quarters (groups 1–3) by farm (farm A and B). All discriminant analyses were performed in JMP Pro (SAS Institute Inc. North Carolina) using the bacterial genus prevalence in each sample as covariates and the sample group (I, II, III) or farm (IV) as the categorical variable. Only genera significant for the discrimination were included in the final models. Groups are colour coded in each figure. The center of gravity for each group is represented by a + sign and variability by a circle.

Discriminant analyses of milk samples microbiome by different groups of samples or by farm are presented in Figure 2. Discriminant analysis was performed using all 5 groups of milk samples, after excluding samples derived from clinical mastitis quarters (groups 1–4) or using only healthy quarters (groups 1–3). Discriminant analysis of the microbiome of milk samples from healthy milk samples (groups 1–3) by farm is also presented in Figure 2.

**Figure 3:**
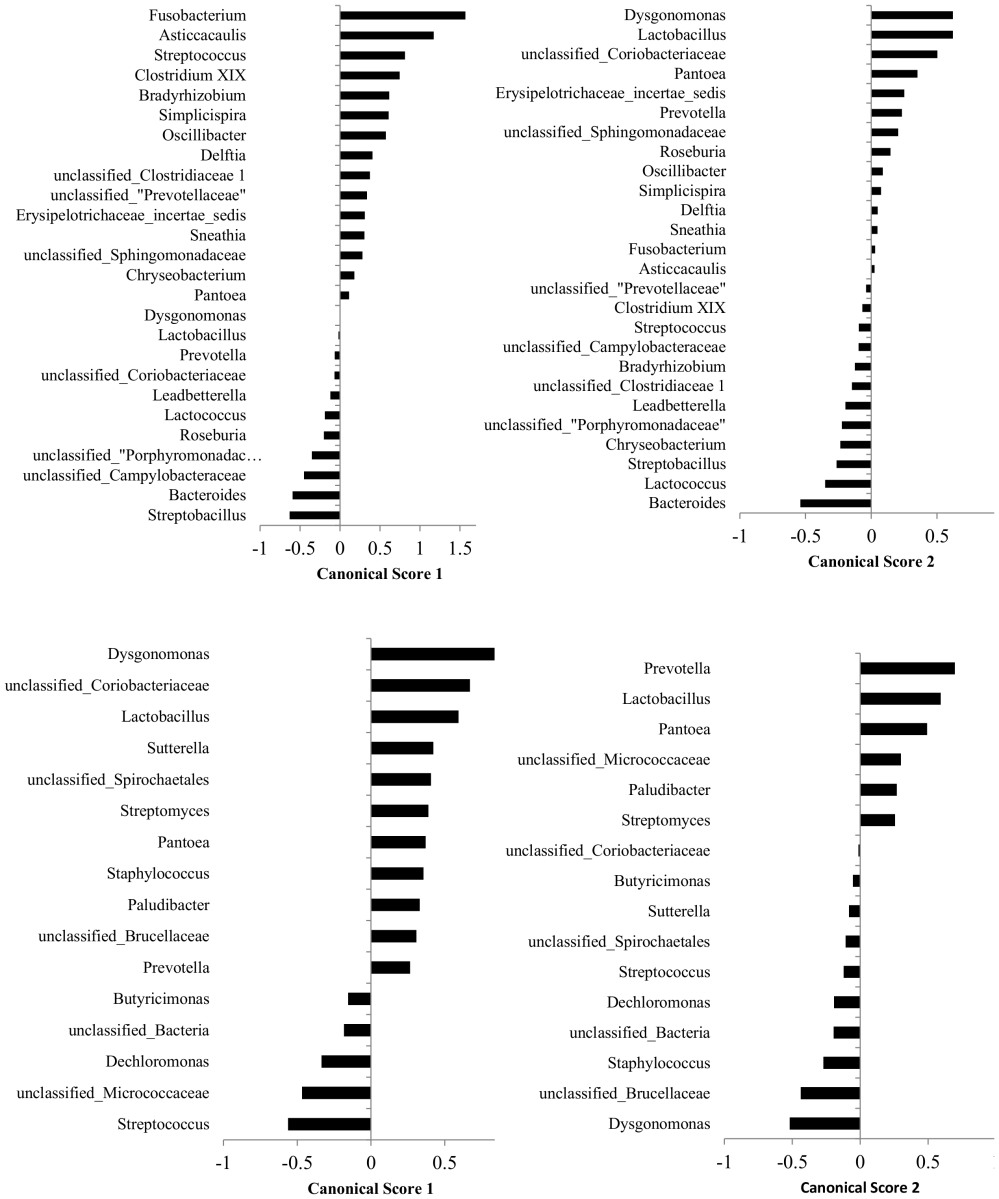
Canonical scores 1 and 2 for genera that were found to be significant for the discriminant analysis (displayed in Figure 2I) of milk microbiome by milk samples groups performed using all 5 groups (top) and canonical scores 1 and 2 for genera that were found to be significant for the discriminant analysis (displayed in Figure 2II) of milk microbiome by milk samples group 1 to 4, excluding group 5 (bottom).

**Figure 4:**
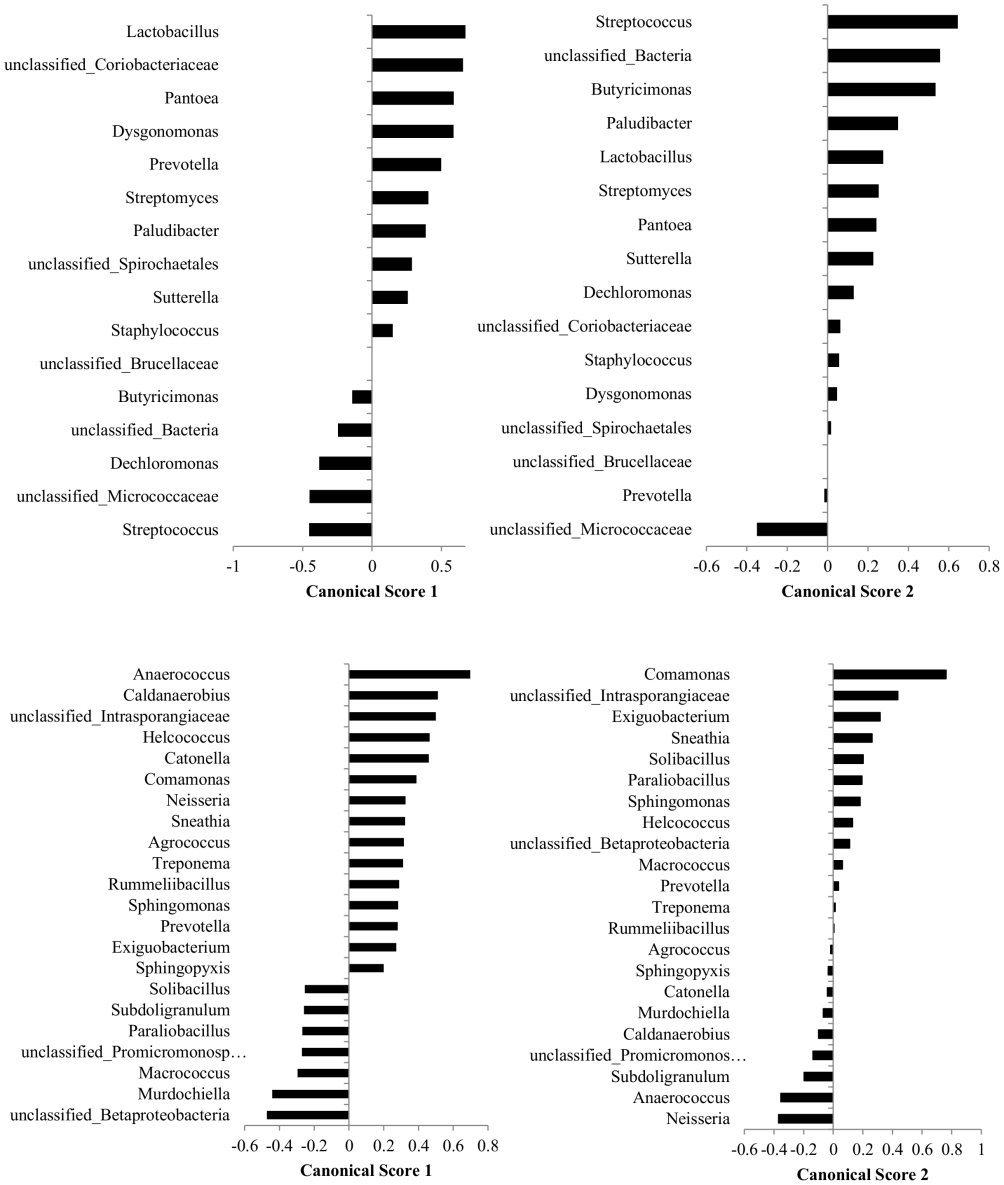
Canonical scores 1 and 2 for genera that were found to be significant for the discriminant analysis (displayed in Figure 2III) of milk microbiome by milk samples groups performed using groups 1–3 (top). Canonical scores 1 and for genera that were found to be significant for the discriminant analysis (displayed in Figure 2IV) of milk microbiome that used farm as the categorical variable and genera prevalences for the milk samples from groups 1–3 (bottom).

Canonical scores 1 and 2 for genera that were found to be significant for the discriminant analysis of milk microbiome by different milk samples groups performed using all 5 groups of milk samples and for genera that were found to be significant for the discriminant analysis of milk microbiome by different groups performed after excluding samples derived from clinical mastitis quarters are presented in Figure 3. Canonical scores 1 and 2 for genera that were found to be significant for the discriminant analysis of milk microbiome by group performed using groups 1–3 and canonical scores 1 and 2 for genera that were found to be significant for the discriminant analysis of milk microbiome that used farm as the categorical variable and genera prevalences for the milk samples from groups 1–3 are presented in Figure 4.

Average prevalences of bacterial genera that were found to be significant for the four different discriminant analysis models are presented in Figures S1, S2, S3, S4 in the supplemental material.

### Generalized multivariable linear model results

**Figure 5:**
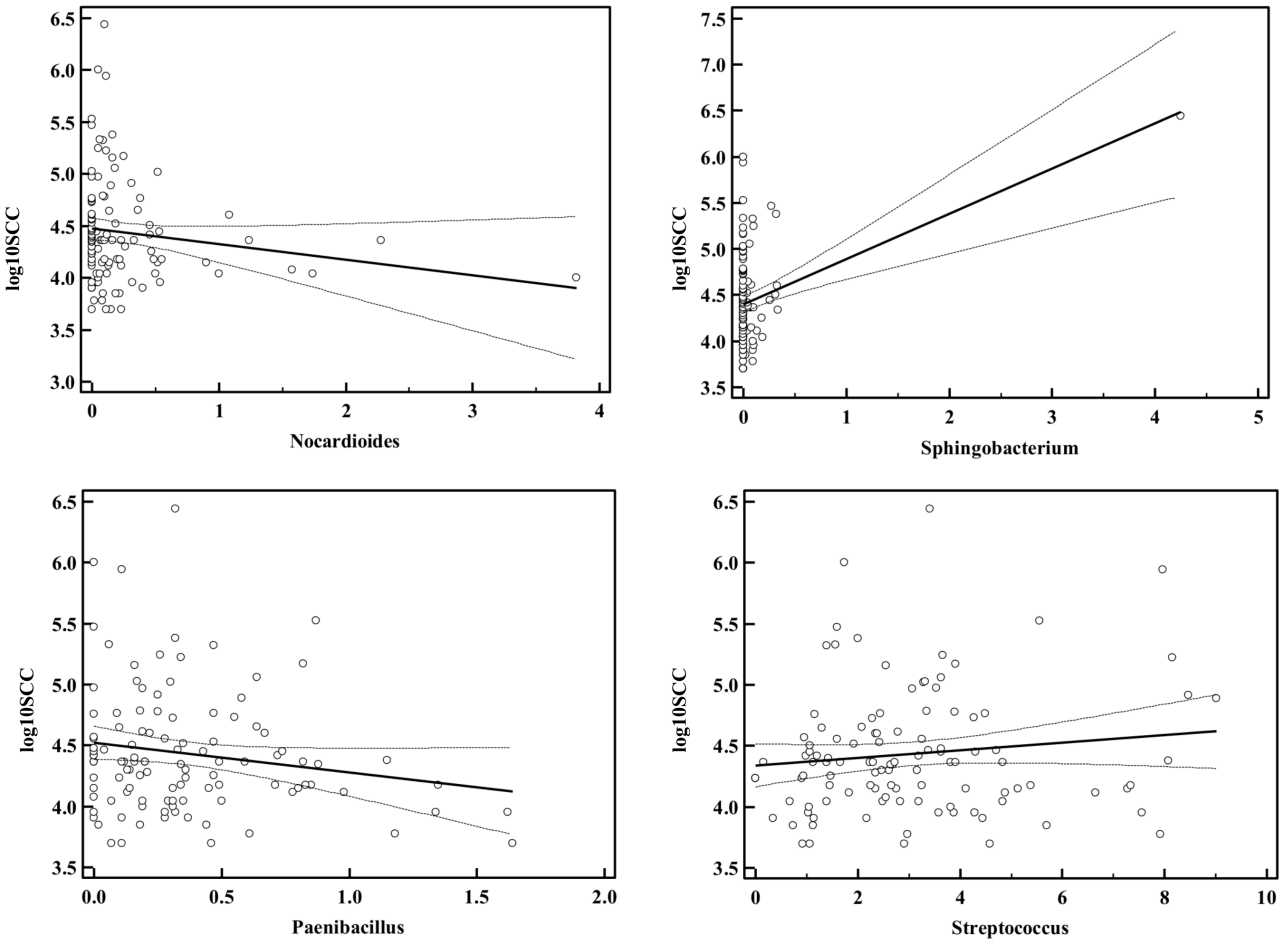
Genera prevalence by log10 somatic cell counts (log10SCC) for genera that are associated with an increased or decreased immune response as measured by SCC in healthy quarters; a generalized linear model was used to predict the 10 Log of quarter SCC. Percent of the sequences for each of the predominant genera were used as predictor variables while at the same time correcting for herd effects.

Regression of 10 Log SCC in groups 1–3 resulted in 4 genera being significantly predictive. With two genera, *Sphingobacterium* and *Streptococcus*, a positive relationship was found where a higher percentage of the genera was associated with increased 10 log SCC. Conversely, a higher percentage of *Nocardiodes* and *Paenibacillus* was associated with a lower 10 log SCC (Figure 5).

### Diversity indexes

Shannon and Chao1 indices estimates for a cut-off value of 0.03 by number of sequences analysed per sample are presented in Figure S5 in the supplemental material. Adjusted means (with confidence intervals) of Chao1 and Shannon diversity indexes for each different group of samples are also presented in Figure S5 in the supplemental material. No significant differences were observed between samples obtained from healthy quarters. However, Shannon index was significantly lower in samples derived from clinically affected quarters. Rarefaction curves for each different group of samples and for a cutoff value of 0.03 are presented in Figure S6 in the supplemental material.

## Discussion

All milk samples analysed here revealed great microbial diversity regardless of their SCC status. Previous studies on healthy human and bovine milk samples reported similar results [11], [13], [14]. Four bacterial genera were present in every sample obtained from healthy quarters (*Faecalibacterium*, unclassified *Lachnospiraceae*, *Propionibacterium* and *Aeribacillus*). *Propionibacterium* spp. was also found to be present in all human milk samples analyzed by Hunt et al. [13]. Jost et al. (2013) [23] also reported that *Propionibacterium* spp. as well as bacteria from the *Lachnospiraceae* family were predominant in milk samples obtained from human mammary glands. Our species level analysis indicated that *Propionibacterium acnes* was the most prevalent bacterium in most of our healthy milk samples. Interestingly, Shu et al. (2013) [24], recently reported that fermentation of *Propionibacterium acnes* in the human skin microbiome may play a role in human innate immunity against *Staphylococcus aureus*. In our samples, *Staphylococcus* spp. and *Streptococcus* spp. were among the most prevalent genera in all groups, again similar to results from human and bovine (*Staphylococcus* spp. only) milk samples in previously published studies [11], [13], [14], [23].

The presence of large numbers of bacterial genomes in milk with no evidence of any inflammatory response or chemotaxis of neutrophils (group 1 and probably group 2) would suggest that the immune cells present in the gland recognize these bacteria as ‘self’. It may be hypothesized that early in life a distinction is made between self and non-self, suggesting the presence of hitherto unidentified Peyer’s patches or M-cells in the developing mammary gland [25]. The presence of a microbial flora recognized as self, or commensal, is also essential for other organ systems that are connected to the outside environment such as the intestinal tract, eyes, ears and the uro-genital tract [25], [26] or in the case of ruminants the rumen [27], [28]. Similar to observations from the intestinal tract, the microbiome of the bovine mammary gland also appears to be specific for the primary residence (source farm in the case of dairy cows) [29].

Discriminant analysis models showed that samples derived from healthy quarters were easily discriminated based on their microbiota profiles from samples derived from clinical mastitis, culture negative quarters (Figure 2I). When only healthy culture negative samples were evaluated (groups 1–3), samples that belonged to the second group (healthy, medium SCC) were discriminated from groups 1 and 3 (Figure 2III). This is of interest as quarters with both very low and higher SCC are known to be at increased risk of intramammary infections [30], [31], suggesting the presence of an ‘optimal’ microbiome in the group with SCC values between 21,000 and 50,000 cells/ml. From Figure S3 it can be seen that *Lactobacillus* spp. were more prevalent in group 2 samples and were also significant for the discrimination between group 2 and group 1 and 3 (Figure 4). Some *Lactobacillus* spp., have been reported as capable of inhibiting major mastitis pathogens including *Escherichia* spp. and *Serratia* spp. [32]. Discriminant analysis could also clearly discriminate bacterial communities from samples obtained from different farms (Figure 2IV).

Genera associated with an increased log10SCC were *Sphingobacterium* and *Streptococcus*. In the case of *Sphingobacterium,* a single sample with a high prevalence (4%) resulted in a positive correlation whereas most samples had a very low prevalence (0–0.5%). This pattern may be expected from a true pathogen. In the case of the *Streptococcus* a gentle positive relationship was found across the observed prevalences. Two genera were increased in samples with lower log10SCC, suggesting a beneficial effect. In the case of *Nocardiodes* a few samples with a high prevalence were responsible for the negative correlation, while in the case of *Paenibacillus* a general negative correlation was observed where higher prevalences of *Paenibacillus* were associated with lower log10SCC. Particularly *Paenibacillus* is of interest as it has been associated with both biocontrol of pathogenic bacteria [33] and a decreased shelf life of milk and milk products [34].

The most prevalent bacterial sequences in the culture negative clinical mastitis samples were from *Caulobacter leidyi* in the family *Sphingomonadaceae* as described before [10]. We show here that prevalence of *Caulobacter leidyi* is very low among samples obtained from healthy quarters regardless of their SCC status. Chen et al. (2012) [35] recently reported that *Caulobacter leidyi* should be reclassified to *Sphingomonas leidyi*. Khuan et al. (2013) [11] reported that *Sphingomonas* spp. were predominant (mean prevalence of 20.42%) in clinical mastitis, culture negative samples while prevalence in healthy quarters was significantly lower. It is therefore reasonable to speculate that a *Sphingomonas* spp. bacteria might be associated with mastitis although both studies were cross-sectional studies that do not provide full proof of pathogenicity.

Sequences belonging to *Staphylococcus chromogenes* were abundant only in samples derived from subclinical quarters that had a SCC greater than 400,000 cells/ml. Nineteen of these samples were also found to be aerobic culture positive for *Staphylococcus* spp. Coagulase negative staphylococci have been frequently isolated from clinical or subclinical mastitis milk samples [36], [37].

In a previous study [10] we were able to identify high numbers of anaerobic bacterial sequences in all clinical mastitis samples, regardless of the aerobic culture-based diagnosis. DNA from *Fusobacterium necrophorum* and other anaerobic bacteria that are known pathogens (e.g. *Porphyromonas levii* [38]) was detected in most of these clinical mastitis milk samples. A possible role of certain anaerobic bacteria as opportunistic pathogens in existing mastitis cases was speculated [10]. We show here that prevalence of these anaerobic microorganisms was low in all four groups of healthy and subclinical samples.

The presence of *Streptococcus uberis* sequences in all groups of samples, with a lower prevalence in groups 1–4 was not expected as this bacterial species is generally recognized as a major mastitis pathogen [39]. Similarly, *Staphylococcus aureus* was present in small quantities in healthy quarters with low and medium SCC. Particularly in the milk samples classified in group 1, no evidence is present of any inflammatory response. It may therefore be hypothesized that these bacterial species that are known to exist on the skin or in the intestinal tract of the cow are in small quantities part of the normal bacterial flora of the mammary gland [40], [41]. In the case of clinical mastitis associated with these two pathogens, it was reported that these bacteria dominated the bacterial flora in the milk [10]. Hence, clinical mastitis may in such cases be hypothesized to be more of a dysbacteriosis, rather than a simple primary infection [42]. In contrast, neither *Escherichia coli* nor *Klebsiella* spp were found to be present in the milk of samples from group 1–3. These Gram-negative bacteria were identified in the microbiome of clinical mastitis samples when also present in aerobic culture [10].

The culture-independent metagenomic approach followed here allowed us to obtain information on normal and mastitic milk that cannot be obtained with the use of traditional aerobic culturing approaches or culture independent molecular techniques that target specific bacteria [7], [8]. For example, bacteria like *Fusobacterium necrophorum, Propionibacterium acnes, Lactobacillus* spp. or *Sphingomonas* spp. cannot be aerobically cultured.

The current study presents a cross-sectional description of the milk microbiome in healthy and subclinically and clinically affected quarters. Although this provides a first evaluation of the bacterial flora in milk, more interesting developments may be expected from longitudinal studies or with confirmation of results presented here by quantitative PCR. The dynamics of the milk microbiome in healthy quarters during different phase of early mammary growth, gestation and lactation will provide more insight in the mechanisms that lead to the establishment of a healthy gland. Given that DNA sequencing technology has advanced at an incredible pace in recent years, leading to astonishing decreases in sequencing cost, such culture independent approaches based on next generation sequencing may also eventually provide with powerful diagnostic tools for mastitis and subclinical mastitis.

## Supporting Information

Figure S1 **Average prevalence of bacterial genera (genera with prevalences lower than 1% are presented in the top and genera with prevalences over 1% in the bottom figure) that were found to be significant for the discriminant analysis of milk samples microbiome by milk samples groups performed using all 5 groups of milk samples (1 = healthy quarter, somatic cell count < 20000; 2 = healthy quarter, somatic cell count ranged from 21000 to 50000; 3 = healthy quarter, somatic cell count >50000; 4 = healthy culture positive quarters, somatic cell count>400000; 5 = mastitic culture negative quarters).**
(TIF)

Figure S2 **Average prevalence (genera with prevalences lower than 0.1% are presented in the top and genera with prevalences over 0.1% in the bottom figure) of bacterial genera that were found to be significant for the discriminant analysis of milk samples microbiome by milk samples groups performed excluding samples derived from quarters showing signs of clinical mastitis (1 = healthy quarter, somatic cell count < 20000; 2 = healthy quarter, somatic cell count ranging from 21000 to 50000; 3 = healthy quarter, somatic cell count >50000; 4 = healthy culture positive quarters, somatic cell count>400000).**
(TIF)

Figure S3 **Average prevalence (genera with prevalences lower than 1% are presented in the top and genera with prevalences over 1% in the bottom figure) of bacterial genera that were found to be significant for the discriminant analysis of milk samples microbiome by milk samples groups performed excluding samples derived from quarters showing signs of clinical mastitis or from subclinical culture positive quarters with a somatic cell count>400000 (1 = culture negative quarter, somatic cell count < 20000; 2 = healthy quarter, somatic cell count ranging from 21000 to 50000; 3 = healthy quarter, somatic cell count >50000).**
(TIF)

Figure S4 **Average prevalence (genera with prevalences lower than 1% are presented in the top and genera with prevalences over 1% in the bottom figure) of bacterial genera that were found to be significant for the discriminant analysis of milk samples microbiome that used farm as the categorical variable and genera prevalences for the milk samples from groups 1-3.**
(TIF)

Figure S5 **Chao1 and Shannon diversity indexes by number of sequences analysed per sample (Top).** Adjusted means (with confidence intervals) of Chao1 and Shannon diversity indexes for each different group of samples (1 = healthy quarter, somatic cell count < 20000; 2 = healthy quarter, somatic cell count ranged from 21000 to 50000; 3 = healthy quarter, somatic cell count >50000; 4 = healthy culture positive quarters, somatic cell count>400000; 5 = mastitic culture negative quarters).
(TIF)

Figure S6 **Rarefaction curves of the microbial communities of each different group of samples (1 = healthy quarter, somatic cell count < 20000; 2 = healthy quarter, somatic cell count ranged from 21000 to 50000; 3 = healthy quarter, somatic cell count >50000; 4 = healthy culture positive quarters, somatic cell count>400000; 5 = mastitic culture negative quarters) for a cutoff value of 0.03.A.**
(TIF)

Table S1 **Species level information (with GenBank Accession number and identity match) for the predominant representative sequences in samples obtained from healthy, culture negative quarters and had a somatic cell count lower than 20.000.**
(DOCX)

Table S2 **Species level information (with GenBank Accession number, and identity match) for the predominant representative sequences in samples obtained from healthy, culture negative quarters that had a somatic cell count that ranged from 21,000 to 50.000.**
(DOCX)

Table S3 **Species level information (with GenBank Accession number, and identity match) for the predominant representative sequences in culture samples obtained from healthy, culture negative quarters that had a SCC greater than 50.000.**
(DOCX)

Table S4 **Species level information (with GenBank Accession number, and identity match) for the predominant representative sequences in samples obtained from healthy culture positive quarters that had a somatic cell count greater than 400.000.**
(DOCX)

Table S5 **Species level information (with GenBank Accession number, and identity match) for the predominant representative sequences in culture negative samples obtained from mastitic quarters.**
(DOCX)

## References

[1] Grohn YT, Gonzalez RN, Wilson DJ, Hertl JA, Bennett G, et al (2005) Effect of pathogen-specific clinical mastitis on herd life in two New York state dairy herds. Prev Vet Med 71: 105–125. 16111778 10.1016/j.prevetmed.2005.06.002

[2] Bar D, Tauer LW, Bennett G, Gonzalez RN, Hertl JA, et al (2008) The cost of generic clinical mastitis in dairy cows as estimated by using dynamic programming. J Dairy Sci 91: 2205–2214. 18487643 10.3168/jds.2007-0573

[3] Hertl JA, Grohn YT, Leach JD, Bar D, Bennett GJ, et al (2010) Effects of clinical mastitis caused by gram-positive and gram-negative bacteria and other organisms on the probability of conception in New York state Holstein dairy cows. J Dairy Sci 93: 1551–1560. 20338432 10.3168/jds.2009-2599

[4] Schepers AJ, Lam TJ, Schukken YH, Wilmink JB, Hanekamp WJ (1997) Estimation of variance components for somatic cell counts to determine thresholds for uninfected quarters. J Dairy Sci 80: 1833–1840. 9276824 10.3168/jds.S0022-0302(97)76118-6

[5] Schukken YH, Wilson DJ, Welcome F, Garrison-Tikofsky L, Gonzalez RN (2003) Monitoring udder health and milk quality using somatic cell counts. Vet Res 34: 579–596. 14556696 10.1051/vetres:2003028

[6] Norman HD, Miller RH, Wright JR, Wiggans GR (2000) Herd and state means for somatic cell count from dairy herd improvement. J Dairy Sci 83: 2782–2788. 11132847 10.3168/jds.S0022-0302(00)75175-7

[7] Koskinen MT, Wellenberg GJ, Sampimon OC, Holopainen J, Rothkamp A, et al (2010) Field comparison of real-time polymerase chain reaction and bacterial culture for identification of bovine mastitis bacteria. J Dairy Sci 93: 5707–5715. 21094742 10.3168/jds.2010-3167

[8] Shome BR, Das Mitra S, Bhuvana M, Krithiga N, Velu D, et al (2011) Multiplex PCR assay for species identification of bovine mastitis pathogens. J Appl Microbiol 111: 1349–1356. 21972842 10.1111/j.1365-2672.2011.05169.x

[9] Bradley AJ, Leach KA, Breen JE, Green LE, Green MJ (2007) Survey of the incidence and aetiology of mastitis on dairy farms in England and Wales. Vet Rec 160: 253–257. 17322356 10.1136/vr.160.8.253

[10] Oikonomou G, Machado VS, Santisteban C, Schukken YH, Bicalho RC (2012) Microbial diversity of bovine mastitic milk as described by pyrosequencing of metagenomic 16s rDNA. PLoS One 7: e47671. 23082192 10.1371/journal.pone.0047671 PMC3474744

[11] Kuehn JS, Gorden PJ, Munro D, Rong R, Dong Q, et al (2013) Bacterial community profiling of milk samples as a means to understand culture-negative bovine clinical mastitis. PLoS One 8: e61959. 23634219 10.1371/journal.pone.0061959 PMC3636265

[12] Boyer PJ (1997) Outbreak of clinical mastitis in dairy cows following 'blitz' therapy. Vet Rec 141: 55. 9253837

[13] Hunt KM, Foster JA, Forney LJ, Schutte UM, Beck DL, et al (2011) Characterization of the diversity and temporal stability of bacterial communities in human milk. PLoS One 6: e21313. 21695057 10.1371/journal.pone.0021313 PMC3117882

[14] Cabrera-Rubio R, Collado MC, Laitinen K, Salminen S, Isolauri E, et al (2012) The human milk microbiome changes over lactation and is shaped by maternal weight and mode of delivery. Am J Clin Nutr 96: 544–551. 22836031 10.3945/ajcn.112.037382

[15] Green MJ, Green LE, Schukken YH, Bradley AJ, Peeler EJ, et al (2004) Somatic cell count distributions during lactation predict clinical mastitis. J Dairy Sci 87: 1256–1264. 15290974 10.3168/jds.S0022-0302(04)73276-2

[16] Ravel J, Gajer P, Abdo Z, Schneider GM, Koenig SS, et al (2011) Vaginal microbiome of reproductive-age women. Proc Natl Acad Sci U S A 108 Suppl 14680–4687. 20534435 10.1073/pnas.1002611107 PMC3063603

[17] Hamady M, Walker JJ, Harris JK, Gold NJ, Knight R (2008) Error-correcting barcoded primers for pyrosequencing hundreds of samples in multiplex. Nat Methods 5: 235–237. 18264105 10.1038/nmeth.1184 PMC3439997

[18] Rozen S, Skaletsky H (2000) Primer3 on the WWW for general users and for biologist programmers. Methods Mol Biol 132: 365–386. 10547847 10.1385/1-59259-192-2:365

[19] Wright ES, Yilmaz LS, Noguera DR (2012) DECIPHER, a search-based approach to chimera identification for 16S rRNA sequences. Appl Environ Microbiol 78: 717–725. 22101057 10.1128/AEM.06516-11 PMC3264099

[20] Wang Q, Garrity GM, Tiedje JM, Cole JR (2007) Naive bayesian classifier for rapid assignment of rRNA sequences into the new bacterial taxonomy. Appl Environ Microbiol 73: 5261–5267. 17586664 10.1128/AEM.00062-07 PMC1950982

[21] Nawrocki EP, Eddy SR (2007) Query-dependent banding (QDB) for faster RNA similarity searches. PLoS Comput Biol 3: e56. 17397253 10.1371/journal.pcbi.0030056 PMC1847999

[22] Altschul SF, Gish W, Miller W, Myers EW, Lipman DJ (1990) Basic local alignment search tool. J Mol Biol 215: 403–410. 2231712 10.1016/S0022-2836(05)80360-2

[23] Jost T, Lacroix C, Braegger C, Chassard C (2013) Assessment of bacterial diversity in breast milk using culture-dependent and culture-independent approaches. Br J Nutr : 1–10. 10.1017/S0007114513000597 23507238

[24] Shu M, Wang Y, Yu J, Kuo S, Coda A, et al (2013) Fermentation of *Propionibacterium acnes*, a commensal bacterium in the human skin microbiome, as skin probiotics against methicillin-resistant staphylococcus aureus. PLoS One 8: e55380. 23405142 10.1371/journal.pone.0055380 PMC3566139

[25] Eberl G, Lochner M (2009) The development of intestinal lymphoid tissues at the interface of self and microbiota. Mucosal Immunol 2: 478–485. 19741595 10.1038/mi.2009.114

[26] Martin R, Nauta AJ, Ben Amor K, Knippels LM, Knol J, et al (2010) Early life: Gut microbiota and immune development in infancy. Benef Microbes 1: 367–382. 21831776 10.3920/BM2010.0027

[27] Li M, Zhou M, Adamowicz E, Basarab JA, Guan LL (2012) Characterization of bovine ruminal epithelial bacterial communities using 16S rRNA sequencing, PCR-DGGE, and qRT-PCR analysis. Vet Microbiol 155: 72–80. 21890283 10.1016/j.vetmic.2011.08.007

[28] Fouts DE, Szpakowski S, Purushe J, Torralba M, Waterman RC, et al (2012) Next generation sequencing to define prokaryotic and fungal diversity in the bovine rumen. PLoS One 7: e48289. 23144861 10.1371/journal.pone.0048289 PMC3492333

[29] Lepage P, Leclerc MC, Joossens M, Mondot S, Blottiere HM, et al (2013) A metagenomic insight into our gut's microbiome. Gut 62: 146–158. 22525886 10.1136/gutjnl-2011-301805

[30] Peeler EJ, Green MJ, Fitzpatrick JL, Green LE (2003) The association between quarter somatic-cell counts and clinical mastitis in three british dairy herds. Prev Vet Med 59: 169–180. 12809761 10.1016/s0167-5877(03)00076-x

[31] Suriyasathaporn W, Schukken YH, Nielen M, Brand A (2000) Low somatic cell count: A risk factor for subsequent clinical mastitis in a dairy herd. J Dairy Sci 83: 1248–1255. 10877390 10.3168/jds.S0022-0302(00)74991-5

[32] Jara S, Sanchez M, Vera R, Cofre J, Castro E (2011) The inhibitory activity of *Lactobacillus* spp. isolated from breast milk on gastrointestinal pathogenic bacteria of nosocomial origin. Anaerobe 17: 474–477. 21846506 10.1016/j.anaerobe.2011.07.008

[33] Debois D, Ongena M, Cawoy H, De Pauw E (2013) MALDI-FTICR MS imaging as a powerful tool to identify *Paenibacillus* antibiotics involved in the inhibition of plant pathogens. J Am Soc Mass Spectrom. 10.1007/s13361-013-0620-2 23636858

[34] Ranieri ML, Ivy RA, Mitchell WR, Call E, Masiello SN, et al (2012) Real-time PCR detection of paenibacillus spp. in raw milk to predict shelf life performance of pasteurized fluid milk products. Appl Environ Microbiol 78: 5855–5863. 22685148 10.1128/AEM.01361-12 PMC3406147

[35] Chen H, Jogler M, Rohde M, Klenk HP, Busse HJ, et al (2012) Reclassification and emended description of *Caulobacter leidyi* as *Sphingomonas leidyi* comb. nov., and emendation of the genus *Sphingomonas* . Int J Syst Evol Microbiol 62: 2835–2843. 22228660 10.1099/ijs.0.039636-0

[36] Pyorala S, Taponen S (2009) Coagulase-negative staphylococci-emerging mastitis pathogens. Vet Microbiol 134: 3–8. 18848410 10.1016/j.vetmic.2008.09.015

[37] Luthje P, Schwarz S (2006) Antimicrobial resistance of coagulase-negative staphylococci from bovine subclinical mastitis with particular reference to macrolide-lincosamide resistance phenotypes and genotypes. J Antimicrob Chemother 57: 966–969. 16524893 10.1093/jac/dkl061

[38] Elad D, Friedgut O, Alpert N, Stram Y, Lahav D, et al (2004) Bovine necrotic vulvovaginitis associated with *Porphyromonas levii* . Emerg Infect Dis 10: 505–507. 15109423 10.3201/eid1003.020592 PMC3322791

[39] Zadoks RN, Middleton JR, McDougall S, Katholm J, Schukken YH (2011) Molecular epidemiology of mastitis pathogens of dairy cattle and comparative relevance to humans. J Mammary Gland Biol Neoplasia 16: 357–372. 21968538 10.1007/s10911-011-9236-y PMC3208832

[40] Zadoks RN, Tikofsky LL, Boor KJ (2005) Ribotyping of *Streptococcus uberis* from a dairy's environment, bovine feces and milk. Vet Microbiol 109: 257–265. 15967600 10.1016/j.vetmic.2005.05.008

[41] Roberson JR, Fox LK, Hancock DD, Gay JM, Besser TE (1994) Ecology of *Staphylococcus aureus* isolated from various sites on dairy farms. J Dairy Sci 77: 3354–3364. 7814712 10.3168/jds.S0022-0302(94)77277-5

[42] Fernandez L, Langa S, Martin V, Maldonado A, Jimenez E, et al (2013) The human milk microbiota: Origin and potential roles in health and disease. Pharmacol Res 69: 1–10. 22974824 10.1016/j.phrs.2012.09.001

