# Supplementary figures and images for: Microbiota of Cow’s Milk; Distinguishing Healthy, Sub-Clinically and Clinically Diseased Quarters
Source: PLoS One. 2014 Jan 20;9(1):e85904. doi: 10.1371/journal.pone.0085904 (PMC3896433; doi:10.1371/journal.pone.0085904)

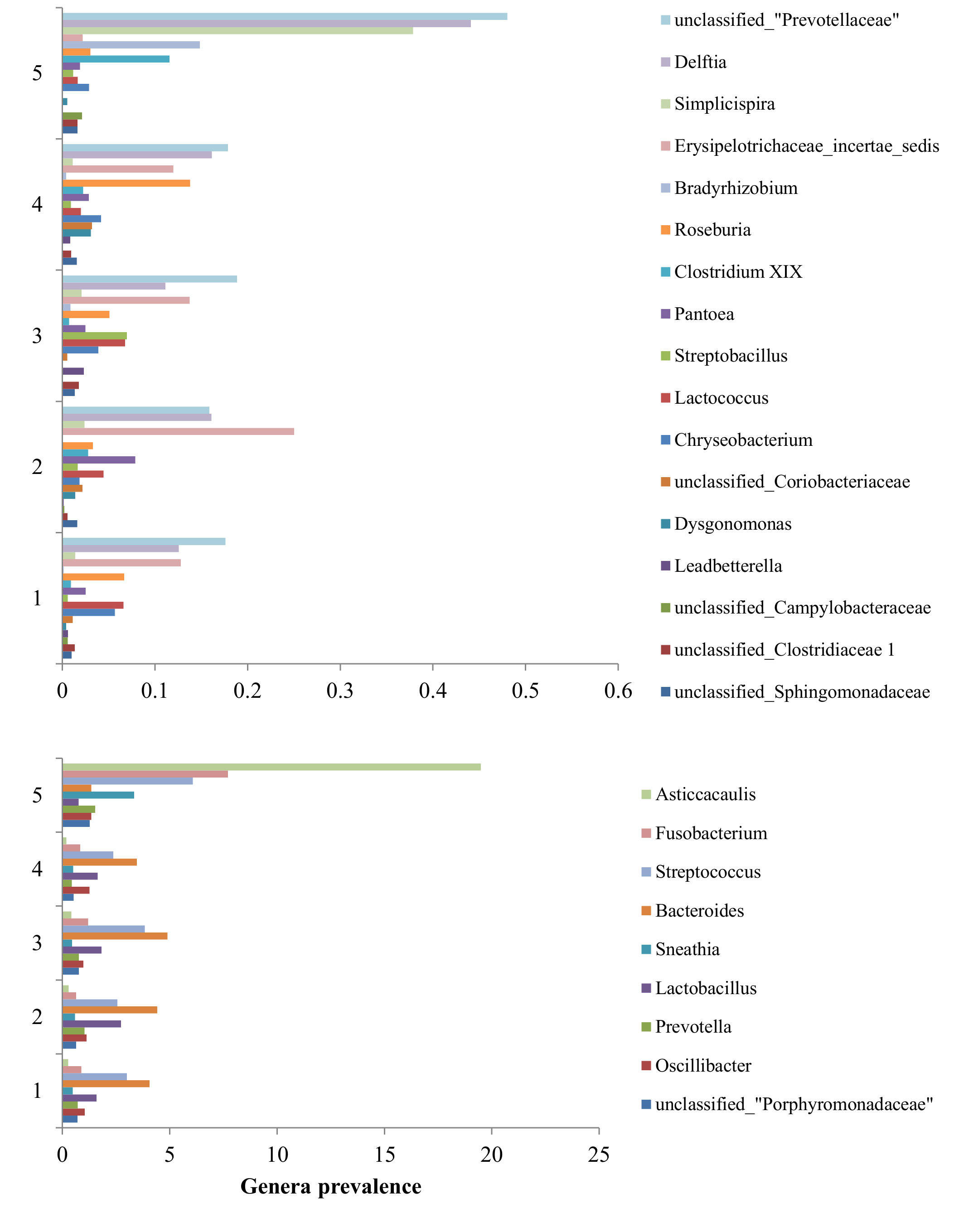

Supplement: Figure S1 — Average prevalence of bacterial genera (genera with prevalences lower than 1% are presented in the top and genera with prevalences over 1% in the bottom figure) that were found to be significant for the discriminant analysis of milk samples microbiome by milk samples groups performed using all 5 groups of milk samples (1 = healthy quarter, somatic cell count < 20000; 2 = healthy quarter, somatic cell count ranged from 21000 to 50000; 3 = healthy quarter, somatic cell count >50000; 4 = healthy culture positive quarters, somatic cell count>400000; 5 = mastitic culture negative quarters). (TIF) [file pone.0085904.s001.tif]

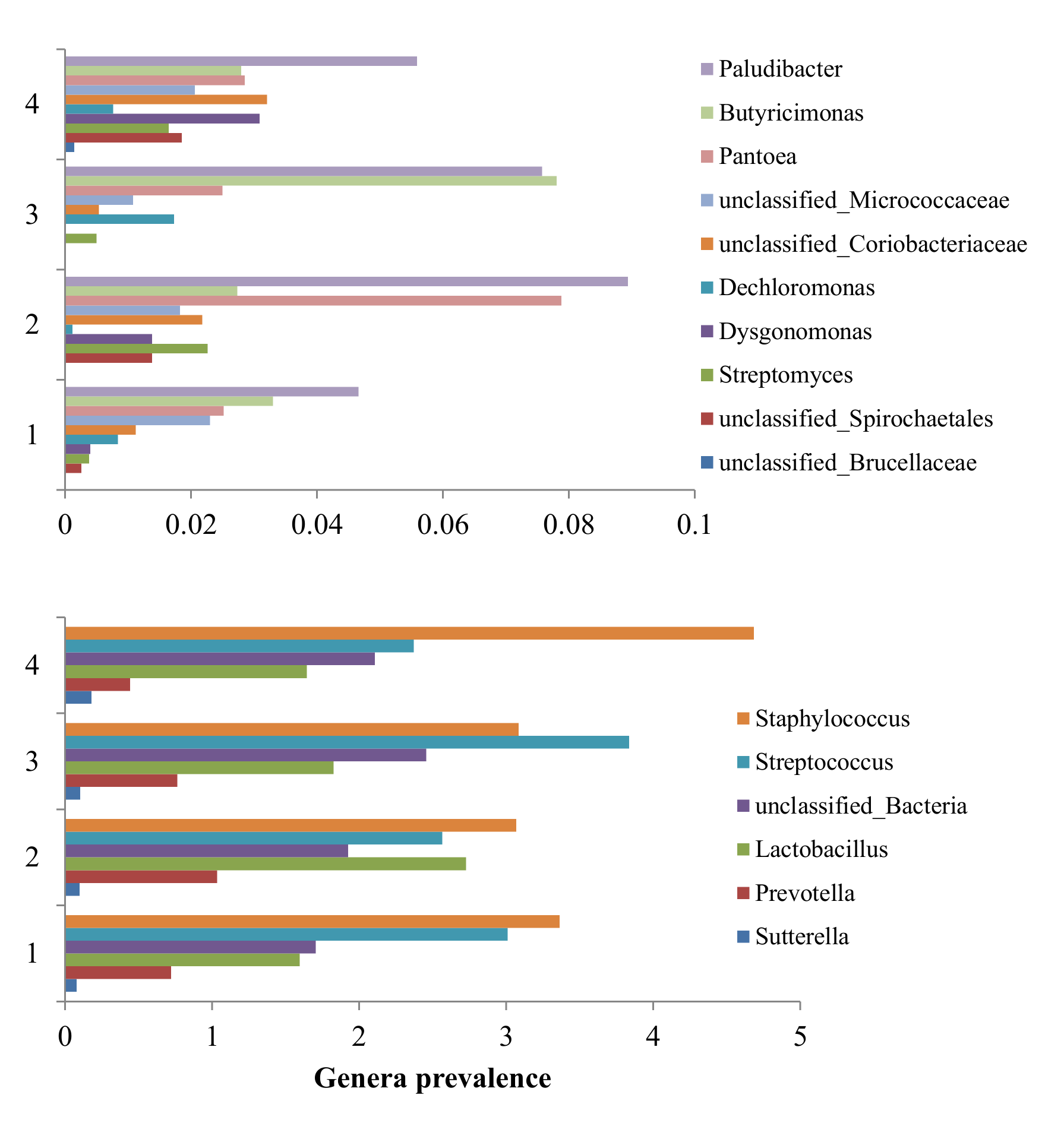

Supplement: Figure S2 — Average prevalence (genera with prevalences lower than 0.1% are presented in the top and genera with prevalences over 0.1% in the bottom figure) of bacterial genera that were found to be significant for the discriminant analysis of milk samples microbiome by milk samples groups performed excluding samples derived from quarters showing signs of clinical mastitis (1 = healthy quarter, somatic cell count < 20000; 2 = healthy quarter, somatic cell count ranging from 21000 to 50000; 3 = healthy quarter, somatic cell count >50000; 4 = healthy culture positive quarters, somatic cell count>400000). (TIF) [file pone.0085904.s002.tif]

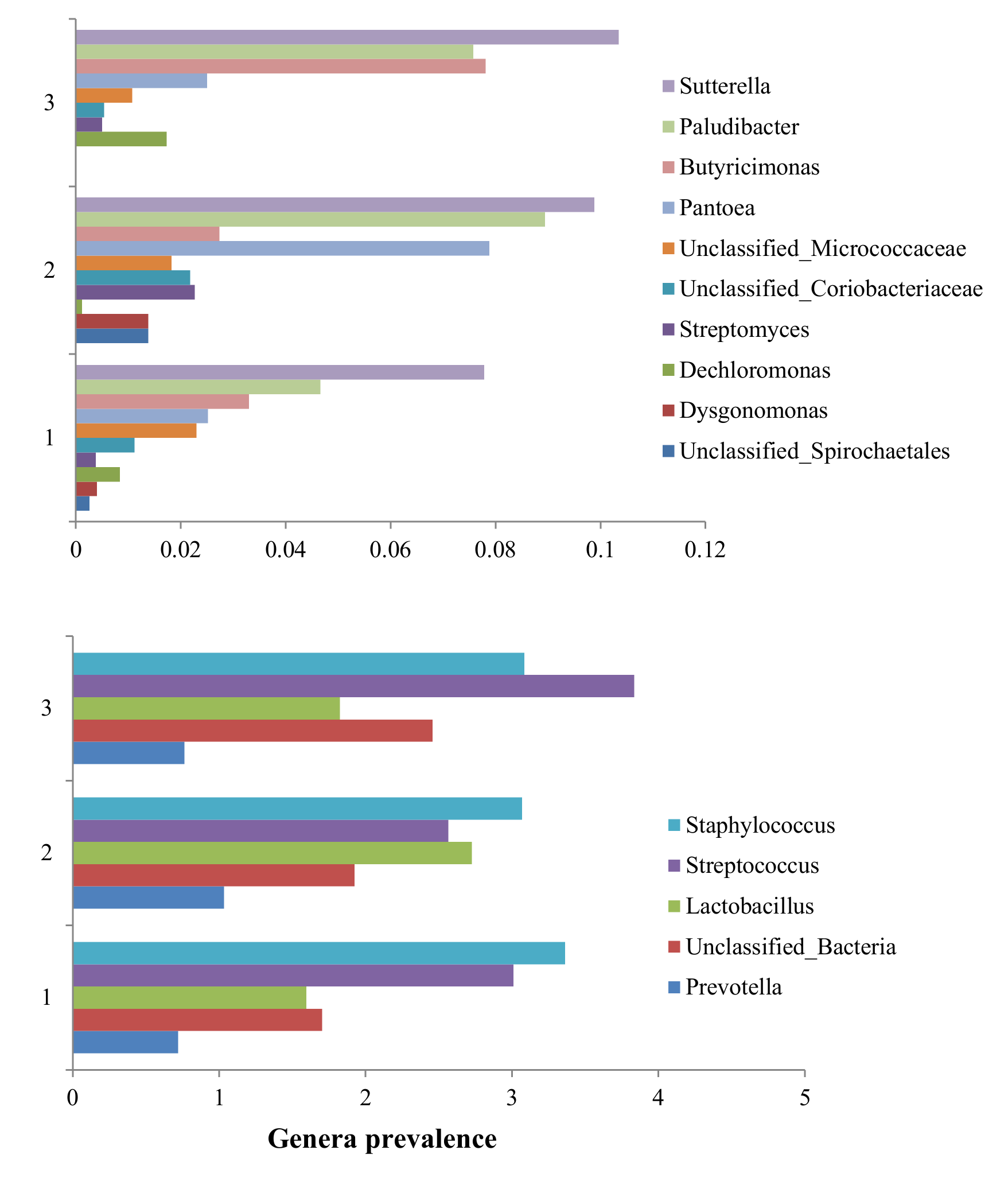

Supplement: Figure S3 — Average prevalence (genera with prevalences lower than 1% are presented in the top and genera with prevalences over 1% in the bottom figure) of bacterial genera that were found to be significant for the discriminant analysis of milk samples microbiome by milk samples groups performed excluding samples derived from quarters showing signs of clinical mastitis or from subclinical culture positive quarters with a somatic cell count>400000 (1 = culture negative quarter, somatic cell count < 20000; 2 = healthy quarter, somatic cell count ranging from 21000 to 50000; 3 = healthy quarter, somatic cell count >50000). (TIF) [file pone.0085904.s003.tif]

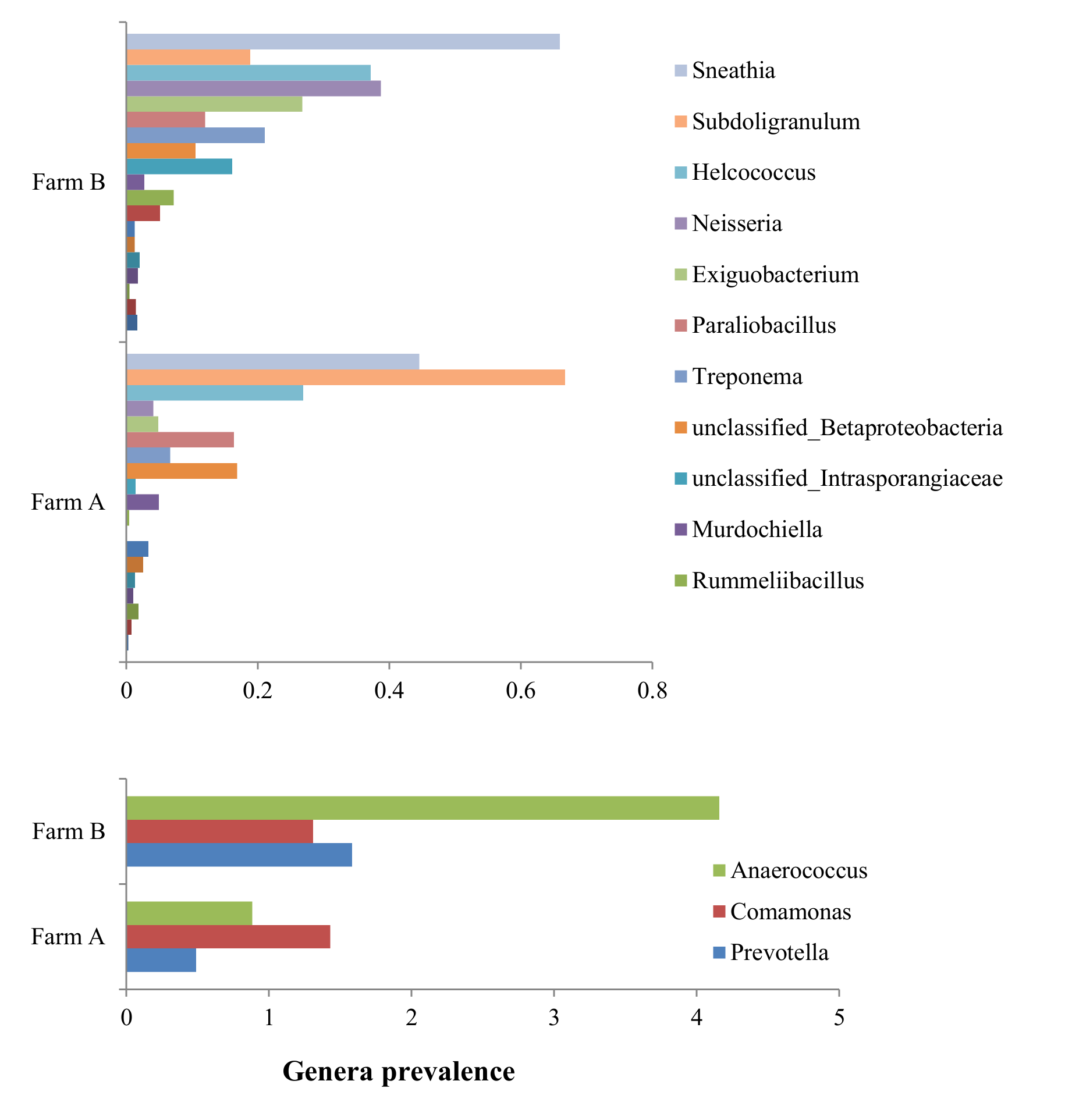

Supplement: Figure S4 — Average prevalence (genera with prevalences lower than 1% are presented in the top and genera with prevalences over 1% in the bottom figure) of bacterial genera that were found to be significant for the discriminant analysis of milk samples microbiome that used farm as the categorical variable and genera prevalences for the milk samples from groups 1-3. (TIF) [file pone.0085904.s004.tif]

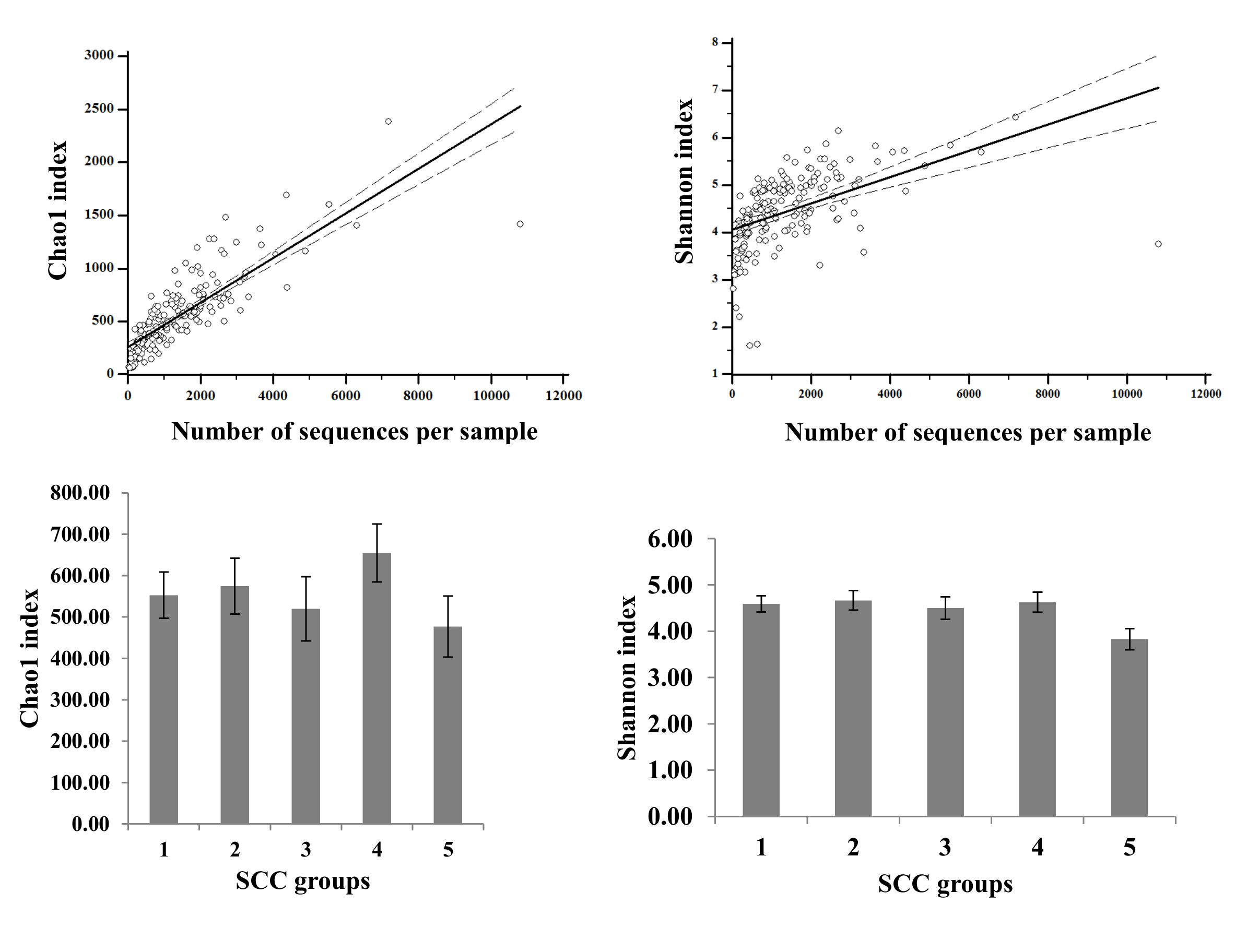

Supplement: Figure S5 — Chao1 and Shannon diversity indexes by number of sequences analysed per sample (Top). Adjusted means (with confidence intervals) of Chao1 and Shannon diversity indexes for each different group of samples (1 = healthy quarter, somatic cell count < 20000; 2 = healthy quarter, somatic cell count ranged from 21000 to 50000; 3 = healthy quarter, somatic cell count >50000; 4 = healthy culture positive quarters, somatic cell count>400000; 5 = mastitic culture negative quarters). (TIF) [file pone.0085904.s005.tif]

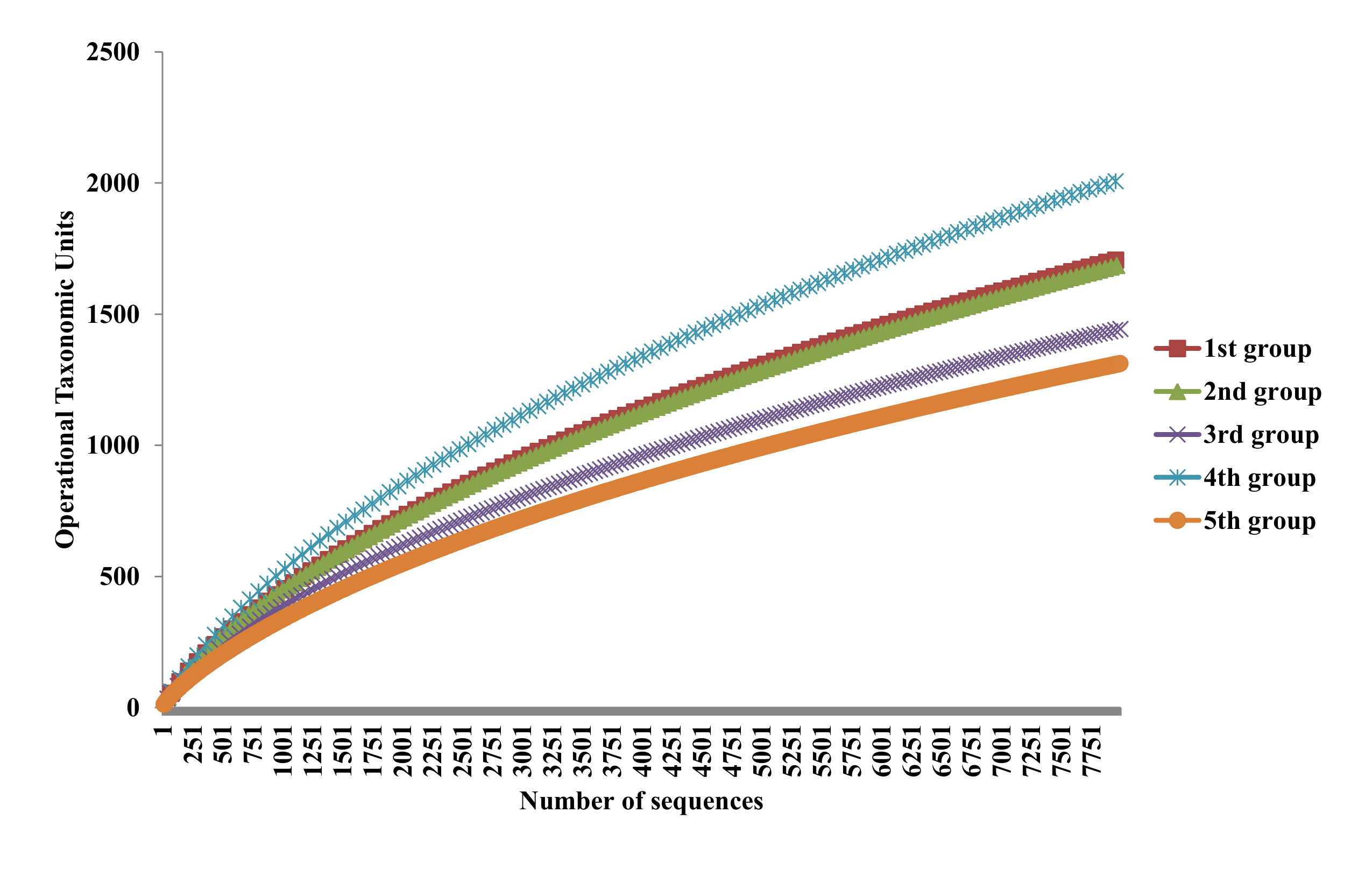

Supplement: Figure S6 — Rarefaction curves of the microbial communities of each different group of samples (1 = healthy quarter, somatic cell count < 20000; 2 = healthy quarter, somatic cell count ranged from 21000 to 50000; 3 = healthy quarter, somatic cell count >50000; 4 = healthy culture positive quarters, somatic cell count>400000; 5 = mastitic culture negative quarters) for a cutoff value of 0.03.A. (TIF) [file pone.0085904.s006.tif]
