# Supplementary material for: Microbiota of Cow’s Milk; Distinguishing Healthy, Sub-Clinically and Clinically Diseased Quarters
Source: PLoS One. 2014 Jan 20;9(1):e85904. doi: 10.1371/journal.pone.0085904 (PMC3896433; doi:10.1371/journal.pone.0085904)
Supplement: Table S1 — Species level information (with GenBank Accession number and identity match) for the predominant representative sequences in samples obtained from healthy, culture negative quarters and had a somatic cell count lower than 20.000. (DOCX) [file pone.0085904.s007.docx]

| Species | Prevalence | Identity (%) | Asseccion No |
| --- | --- | --- | --- |
| *Propionibacterium acnes* | 11.37 | 100 | NR_074675.1 |
| *Geobacillus pallidus* | 8.98 | 99 | NR_026515.1 |
| *Streptococcus uberis* | 2.03 | 100 | NR_074912.1 |
| *Staphylococcus epidermidis* | 1.82 | 100 | NR_036904.1 |
| Uncultured *Firmicutes* | 1.50 | 100 | KC169766.1 |
| *Comamonas kerstersii* | 1.32 | 99 | NR_025530.1 |
| Uncultured bacterium | 1.30 | 99 | JX634191.1 |
| Uncultured bacterium | 2.33 | 99 | JX632191.1 |
| *Bacteroides coprophilus* | 1.13 | 99 | NR_041461.1 |
| Uncultured bacterium | 1.01 | 100 | JX634861.1 |
| *Bacteroides vulgatus* | 0.94 | 99 | NR_074515.1 |
| *Faecalibacterium prausnitzii* | 0.82 | 98 | NR_028961.1 |
| *Bacteroides heparinolyticus* | 0.81 | 99 | NR_044633.1 |
| Uncultured bacterium | 0.74 | 99 | JX633912.1 |
| Uncultured bacterium clone | 0.72 | 99 | EU772991.1 |
| *Lactobacillus johnsonii* | 0.71 | 100 | AB809591.1 |
| Uncultured bacterium | 0.70 | 99 | JX631380.1 |
| Uncultured *Clostridiales bacterium* | 0.65 | 99 | JQ083415.1 |
| Uncultured *Bacteroides* | 0.63 | 99 | JN167632.1 |
| Uncultured *Porphyromonas* | 0.58 | 99 | JN167617.1 |
| *Staphylococcus equorum* | 0.58 | 100 | JX154400.1 |
| Uncultured *bacterium* | 0.56 | 99 | HE576074.1 |
| Uncultured *bacterium* | 0.54 | 99 | JQ185500.1 |
| Uncultured *bacterium* | 0.54 | 100 | GQ094318.1 |
| *Bacteroides fragilis* | 0.53 | 100 | NR_074784.1 |
| Uncultured *bacterium* | 0.52 | 100 | JX631909.1 |
| *Fusobacterium necrophorum* | 0.47 | 99 | JN713357.1 |
| *Porphyromonas levii* | 0.46 | 100 | AB547664.1 |
| Uncultured *bacterium* | 0.44 | 100 | JX107154.1 |
| Uncultured *bacterium* | 0.44 | 99 | JX634922.1 |
| *Kocuria* | 0.43 | 99 | KC009524.1 |
| Uncultured bacterium | 0.42 | 99 | JF643239.1 |
| Uncultured bacterium | 0.41 | 100 | JQ377512.2 |
| Uncultured bacterium | 0.39 | 99 | JX634111.1 |
| Uncultured bacterium | 0.38 | 100 | JX109525.1 |
| Uncultured bacterium | 0.36 | 99 | JX634914.1 |
| Uncultured bacterium | 0.36 | 99 | FJ681872.1 |
| Uncultured *Halomonas* | 0.35 | 100 | JX240570.1 |
| *Rhodanobacter* | 0.35 | 100 | FJ821730.1 |
| Uncultured *bacterium* | 0.35 | 100 | JX631814.1 |
| Uncultured *bacterium* | 0.35 | 100 | JQ455637.1 |
| Uncultured *bacterium* | 0.34 | 100 | JX633904.1 |
| *Enterococcus faecalis* | 0.33 | 100 | KC481313.1 |
| Uncultured *bacterium* | 0.32 | 99 | JF194563.1 |
| Uncultured *bacterium* | 0.32 | 99 | JX106677.1 |
| *Staphylococcus aureus* | 0.32 | 100 | NR_075000.1 |
| Uncultured bacterium | 0.31 | 100 | JX108913.1 |
