# Supplementary material for: Microbiota of Cow’s Milk; Distinguishing Healthy, Sub-Clinically and Clinically Diseased Quarters
Source: PLoS One. 2014 Jan 20;9(1):e85904. doi: 10.1371/journal.pone.0085904 (PMC3896433; doi:10.1371/journal.pone.0085904)
Supplement: Table S2 — Species level information (with GenBank Accession number, and identity match) for the predominant representative sequences in samples obtained from healthy, culture negative quarters that had a somatic cell count that ranged from 21,000 to 50.000. (DOCX) [file pone.0085904.s008.docx]

| Species | Prevalence | Identity (%) | Assecion No |
| --- | --- | --- | --- |
| *Propionibacterium acnes* | 9.05 | 99 | CP003293.1 |
| *Geobacillus pallidus* | 8.93 | 99 | HM030740.1 |
| Uncultured bacterium | 2.39 | 100 | JQ186970.1 |
| Uncultured *Bacteroides* | 1.58 | 100 | KC467106.1 |
| *Staphylococcus epidermidis* | 1.57 | 100 | KC443110.1 |
| Uncultured bacterium | 1.55 | 99 | FJ682454.1 |
| *Streptococcus uberis* | 1.42 | 100 | KC510224.1 |
| Uncultured bacterium | 1.34 | 100 | JX634191.1 |
| *Clostridiales bacterium* | 1.24 | 99 | HQ452852.1 |
| *Acidovorax* | 1.12 | 100 | AY258065.1 |
| *Bacteroides vulgatus* | 1.00 | 100 | NR_074515.1 |
| Uncultured *Firmicutes* | 0.95 | 100 | KC169766.1 |
| *Lactobacillus johnsonii* | 0.95 | 100 | AB809591.1 |
| Uncultured bacterium | 0.88 | 100 | JX633912.1 |
| Uncultured bacterium | 0.76 | 99 | KC245471.1 |
| Uncultured bacterium | 0.75 | 100 | EU772991.1 |
| *Lactobacillus reuteri* | 0.75 | 100 | JX272060.1 |
| Uncultured bacterium | 0.70 | 99 | JF110839.1 |
| Uncultured bacterium | 0.61 | 100 | JQ186761.1 |
| Uncultured bacterium | 0.61 | 100 | KC328167.1 |
| Uncultured bacterium | 0.60 | 100 | JF196738.1 |
| Uncultured bacterium | 0.58 | 100 | JX635311.1 |
| Uncultured bacterium | 0.58 | 99 | JX634111.1 |
| Uncultured bacterium | 0.58 | 100 | JX631380.1 |
| uncultured bacterium | 0.55 | 99 | HE576074.1 |
| Uncultured bacterium | 0.55 | 99 | JF643239.1 |
| *Bacteroides fragilis* | 0.54 | 100 | NR_074784.1 |
| *Clostridium* | 0.52 | 99 | AB739698.1 |
| *Staphylococcus equorum* | 0.52 | 100 | JX154400.1 |
| *Porphyromonas levii* | 0.51 | 100 | AB547664.1 |
| Uncultured bacterium | 0.51 | 100 | FJ683355.1 |
| Uncultured bacterium | 0.51 | 99 | JX634922.1 |
| *Lactobacillus acidophilus* | 0.51 | 100 | NR_075049.1 |
| Uncultured bacterium | 0.49 | 100 | JX107493.1 |
| Uncultured bacterium | 0.49 | 100 | FJ681872.1 |
| *Enterococcus faecalis* | 0.49 | 100 | KC481313.1 |
| Uncultured *Porphyromonas* | 0.48 | 99 | JN167617.1 |
| Uncultured *Lachnospiraceae* | 0.46 | 99 | EF698785.1 |
| Uncultured *Bacteroides* | 0.45 | 99 | JN167632.1 |
| Uncultured *bacterium* | 0.43 | 100 | GQ136816.1 |
| *Clostridiales bacterium* | 0.43 | 99 | HQ452852.1 |
| *Bacteroides heparinolyticus* | 0.43 | 100 | GQ422742.1 |
| Uncultured *Lachnospiraceae* | 0.42 | 99 | EF698003.1 |
| Uncultured bacterium | 0.42 | 99 | JX634478.1 |
| *Staphylococcus aureus* | 0.39 | 100 | HF937103.1 |
| Uncultured *Helcococcus* sp. | 0.39 | 100 | JN167606.1 |
| Uncultured bacterium | 0.39 | 100 | JF194563.1 |
| *Fusobacterium necrophorum* | 0.39 | 100 | JN713357.1 |
| Uncultured bacterium | 0.37 | 99 | JX109525.1 |
| *Caulobacter leidyia* | 0.36 | 100 | GQ891702.1 |
| *Rhodanobacter terrae* | 0.36 | 100 | FJ405366.1 |
| *Corynebacterium falsenii* | 0.34 | 100 | AF537594.1 |
| Uncultured *Halomonas* sp. | 0.33 | 100 | JX240570.1 |
| Uncultured *bacterium* | 0.33 | 100 | JX106677.1 |
| *Bacteroides denticanum* | 0.31 | 100 | JN713349.1 |
