# Supplementary material for: Microbiota of Cow’s Milk; Distinguishing Healthy, Sub-Clinically and Clinically Diseased Quarters
Source: PLoS One. 2014 Jan 20;9(1):e85904. doi: 10.1371/journal.pone.0085904 (PMC3896433; doi:10.1371/journal.pone.0085904)
Supplement: Table S3 — Species level information (with GenBank Accession number, and identity match) for the predominant representative sequences in culture samples obtained from healthy, culture negative quarters that had a SCC greater than 50.000. (DOCX) [file pone.0085904.s009.docx]

| Species | Prevalence | Identity (%) | Accession No |
| --- | --- | --- | --- |
| *Propionibacterium acnes* | 13.26 | 99 | CP003293.1 |
| *Geobacillus pallidus* | 6.97 | 100 | FJ808721.1 |
| Uncultured bacterium | 1.97 | 99 | FJ682454.1 |
| *Streptococcus uberis* | 1.88 | 100 | KC510224.1 |
| Uncultured bacterium | 1.67 | 0 | FR874056.0 |
| Uncultured bacterium | 1.66 | 99 | GQ179047.1 |
| Uncultured *Proteobacterium* | 1.58 | 100 | GU956128.1 |
| Uncultured *Bacteroides* | 1.58 | 100 | KC467106.1 |
| *Staphylococcus epidermidis* | 1.51 | 100 | KC443110.1 |
| *Clostridiales* bacterium | 1.45 | 100 | HQ452852.1 |
| Uncultured *Bacteroides* | 1.13 | 100 | KC467151.1 |
| Uncultured bacterium | 0.99 | 100 | JX634191.1 |
| Uncultured bacterium | 0.90 | 99 | JX633912.1 |
| Uncultured bacterium | 0.87 | 100 | JX107154.1 |
| Uncultured bacterium | 0.85 | 99 | EU772991.1 |
| Uncultured *Clostridiales* bacterium | 0.84 | 99 | JQ083415.1 |
| *Lactobacillus johnsonii* | 0.79 | 100 | AB809591.1 |
| *Fusobacterium necrophorum* | 0.71 | 100 | JN713357.1 |
| Uncultured organism | 0.71 | 100 | HQ794421.1 |
| *Kocuria* | 0.66 | 100 | KC009524.1 |
| Uncultured bacterium | 0.66 | 99 | JX107775.1 |
| Uncultured bacterium | 0.66 | 99 | JX108565.1 |
| Uncultured bacterium | 0.60 | 100 | FN658870.1 |
| Uncultured *Porphyromonas* | 0.60 | 100 | JN167617.1 |
| Uncultured bacterium | 0.58 | 99 | GQ094318.1 |
| Uncultured bacterium | 0.57 | 99 | GQ094318.1 |
| *Lactobacillus acidophilus* | 0.54 | 100 | NR_075049.1 |
| *Staphylococcus equorum* | 0.52 | 100 | JX154400.1 |
| *Bacteroides fragilis* | 0.52 | 100 | NR_074784.1 |
| Uncultured bacterium | 0.50 | 99 | HE576074.1 |
| Uncultured bacterium | 0.46 | 100 | JX631814.1 |
| *Porphyromonas levii* | 0.46 | 99 | AB547664.1 |
| *Bacteroides heparinolyticus* | 0.44 | 100 | GQ422742.1 |
| Uncultured bacterium | 0.43 | 100 | JQ455637.1 |
| Uncultured bacterium | 0.43 | 100 | JX632130.1 |
| *Rhodanobacter* | 0.41 | 99 | FJ821730.1 |
| Uncultured Gram-positive bacterium | 0.41 | 100 | AB191022.1 |
| Uncultured bacterium | 0.41 | 100 | JF194563.1 |
| Uncultured bacterium | 0.39 | 100 | JF194563.1 |
| Uncultured bacterium | 0.39 | 99 | JX108757.1 |
| Uncultured bacterium | 0.38 | 99 | JX631380.1 |
| Uncultured bacterium | 0.38 | 99 | GQ449096.1 |
| Uncultured bacterium | 0.38 | 99 | JX634478.1 |
| Uncultured *Staphylococcus* | 0.38 | 100 | GU132084.2 |
| *Bacteroides denticanum* | 0.35 | 100 | JN713349.1 |
| *Caulobacter leidyia* | 0.35 | 100 | GQ891702.1 |
| Uncultured bacterium | 0.35 | 99 | JX634111.1 |
| Uncultured bacterium | 0.33 | 99 | JX108447.1 |
