# Supplementary material for: Microbiota of Cow’s Milk; Distinguishing Healthy, Sub-Clinically and Clinically Diseased Quarters
Source: PLoS One. 2014 Jan 20;9(1):e85904. doi: 10.1371/journal.pone.0085904 (PMC3896433; doi:10.1371/journal.pone.0085904)
Supplement: Table S4 — Species level information (with GenBank Accession number, and identity match) for the predominant representative sequences in samples obtained from healthy culture positive quarters that had a somatic cell count greater than 400.000. (DOCX) [file pone.0085904.s010.docx]

| Species | Prevalence | Identity (%) | Accession No |
| --- | --- | --- | --- |
| *Geobacillus pallidus* | 10.08 | 99 | HM030740.1 |
| *Propionibacterium acnes* | 8.35 | 99 | CP003293.1 |
| *Staphylococcus chromogenes* | 2.27 | 100 | AY126169.1 |
| Uncultured bacterium | 1.73 | 99 | JQ186970.1 |
| Uncultured *Bacteroides* | 1.26 | 100 | KC467106.1 |
| *Streptococcus uberis* | 1.22 | 100 | KC510224.1 |
| *Prevotella* | 1.14 | 100 | FJ848548.1 |
| *Staphylococcus epidermidis* | 1.13 | 100 | KC443110.1 |
| Uncultured *bacterium* | 1.05 | 99 | FJ682049.1 |
| *Clostridiales bacterium* | 0.94 | 100 | HQ452852.1 |
| Uncultured bacterium | 0.81 | 100 | JX634191.1 |
| Uncultured *Clostridiales* | 0.75 | 99 | JQ083415.1 |
| Uncultured bacterium | 0.73 | 100 | AB596952.1 |
| Uncultured bacterium | 0.70 | 99 | HM278682.1 |
| *Lactobacillus johnsonii* | 0.68 | 99 | AB809591.1 |
| Uncultured bacterium | 0.67 | 99 | HE576074.1 |
| Uncultured bacterium | 0.65 | 100 | EU772991.1 |
| Uncultured *Bacteroides* | 0.54 | 100 | KC467151.1 |
| *Staphylococcus* | 0.54 | 100 | AB680516.1 |
| Uncultured *Helcococcus* | 0.54 | 100 | JN167606.1 |
| Uncultured bacterium | 0.52 | 99 | JX633912.1 |
| Uncultured bacterium | 0.52 | 100 | JX632130.1 |
| *Piscibacillus* | 0.49 | 96 | GQ169533.1 |
| *Lactobacillus reuteri* | 0.48 | 99 | JX272059.1 |
| *Staphylococcus equorum* | 0.46 | 100 | JX154400.1 |
| *Fusobacterium necrophorum* | 0.43 | 100 | JQ299277.1 |
| Uncultured bacterium | 0.41 | 100 | JX107493.1 |
| Uncultured bacterium | 0.41 | 100 | JX634349.1 |
| Uncultured bacterium | 0.41 | 100 | JF110839.1 |
| *Porphyromonas levii* | 0.41 | 100 | AB547664.1 |
| Uncultured *Clostridia* | 0.40 | 96 | DQ350807.1 |
| *Bacteroides fragilis* | 0.38 | 100 | NR_074784.1 |
| Uncultured bacterium | 0.38 | 100 | JF194563.1 |
| Uncultured bacterium | 0.37 | 100 | HM296441.1 |
| Uncultured bacterium | 0.37 | 100 | GU608811.1 |
| Uncultured bacterium | 0.35 | 98 | GU612182.1 |
| Uncultured bacterium | 0.35 | 100 | JX635311.1 |
| Uncultured bacterium | 0.33 | 99 | JX634922.1 |
| *Geobacillus* | 0.32 | 100 | CP004008.1 |
| Uncultured bacterium | 0.32 | 96 | GU171247.1 |
| Uncultured *Porphyromonas* | 0.32 | 99 | JN167617.1 |
| Uncultured bacterium | 0.32 | 99 | JX632299.1 |
| *Lactobacillus acidophilus* | 0.30 | 100 | NR_075049.1 |
| Uncultured bacterium | 0.30 | 99 | JX634478.1 |
| Uncultured bacterium | 0.30 | 99 | JQ185500.1 |
| *Clostridiales bacterium* | 0.30 | 99 | HQ452852.1 |
