# Supplementary material for: Microbiota of Cow’s Milk; Distinguishing Healthy, Sub-Clinically and Clinically Diseased Quarters
Source: PLoS One. 2014 Jan 20;9(1):e85904. doi: 10.1371/journal.pone.0085904 (PMC3896433; doi:10.1371/journal.pone.0085904)
Supplement: Table S5 — Species level information (with GenBank Accession number, and identity match) for the predominant representative sequences in culture negative samples obtained from mastitic quarters. (DOCX) [file pone.0085904.s011.docx]

| Species | Prevalence | Identity (%) | Accession No |
| --- | --- | --- | --- |
| *Caulobacter leidyia* | 20.55 | 98.9 | GQ891704 |
| Uncultured *Fusobacteria* | 5.49 | 100 | EF704825 |
| *Geobacillus pallidus* | 5.09 | 100 | FJ808716 |
| *Streptococcus uberis* | 4.82 | 100 | HQ326694 |
| Uncultured bacterium | 3.32 | 100 | JF643239 |
| *Propionibacterium acnes* | 2.76 | 100 | CP002409 |
| Uncultured bacterium | 2.46 | 100 | EU289919 |
| *Porphyromonas levii* | 2.09 | 100 | AB547664 |
| Uncultured *Porphyromonas* | 1.46 | 99 | HM754526 |
| *Staphylococcus equorum* | 1.40 | 99.7 | AB334773 |
| Swine manure | 1.33 | 99.7 | AF445295 |
| *Bacteroides heparinolyticus* | 1.30 | 100 | GQ422742 |
| *Ureaplasma diversum* | 1.23 | 99 | NR_025878 |
| *Paenibacillus borealis* | 1.03 | 99.4 | HM563046 |
| Uncultured *Porphyromonas* | 1.00 | 100 | HM754526 |
| Uncultured bacterium | 0.96 | 99.7 | EU290110 |
| Uncultured *Prevotella* | 0.96 | 99.2 | GU905978 |
| Uncultured bacterium | 0.70 | 95.6 | AM183009 |
| *Prevotella spp.* | 0.70 | 100 | FJ848548 |
| Uncultured bacterium | 0.66 | 100 | EU290098 |
| *Histophilus somni* | 0.66 | 99 | [AB176902.1](http://www.ncbi.nlm.nih.gov/nucleotide/62122464?report=genbank&log$=nucltop&blast_rank=1&RID=BAMM9CZF013) |
| Uncultured bacterium | 0.63 | 99.3 | AB107461 |
| *Bacillus spp.* | 0.63 | 100 | FR749853 |
| Uncultured bacterium | 0.60 | 100 | EF205694 |
| *Ochrobactrum pseudogrignonense* | 0.57 | 99.6 | FJ859687 |
| *Helcococcus ovis* | 0.53 | 99.7 | NR_027228 |
| *Corynebacterium falsenii* | 0.53 | 100 | AF537594 |
| *Uncultured bacterium* | 0.47 | 99.7 | HM316969 |
| *Trueperella pyogenes* | 0.43 | 100 | JN578133 |
| Uncultured bacterium | 0.40 | 100 | GU601118 |
| *Delftia spp.* | 0.40 | 100 | CP002735 |
| *Escherichia coli* | 0.37 | 100 | CP001671 |
| Uncultured *Ruminococcaceae* | 0.37 | 99.7 | EU794142 |
| *Mycoplasma bovigenitalium* | 0.37 | 99.7 | AY121098 |
| *Xanthomonas campestris* | 0.33 | 100 | CP002789 |
| Uncultured bacterium | 0.33 | 95.1 | HM257593 |
| Uncultured *Bacteroidetes* | 0.33 | 100 | FM252970 |
| *Brevibacillus parabrevis* | 0.33 | 99.7 | JN315628 |
| Uncultured *Staphylococcus* | 0.33 | 100 | JN082690 |
